# Affective computing of multi-type urban public spaces to analyze emotional quality using ensemble learning-based classification of multi-sensor data

**DOI:** 10.1371/journal.pone.0269176

**Published:** 2022-06-03

**Authors:** Ruixuan Li, Takaya Yuizono, Xianghui Li

**Affiliations:** 1 School of Art and Design, Dalian Polytechnic University, Dalian City, Liaoning Province, China; 2 Graduate School of Advanced Science and Technology, Japan Advanced Institute of Science and Technology, Nomi, Ishikawa, Japan; National University of Sciences and Technology (NUST), PAKISTAN

## Abstract

The quality of urban public spaces affects the emotional response of users; therefore, the emotional data of users can be used as indices to evaluate the quality of a space. Emotional response can be evaluated to effectively measure public space quality through affective computing and obtain evidence-based support for urban space renewal. We proposed a feasible evaluation method for multi-type urban public spaces based on multiple physiological signals and ensemble learning. We built binary, ternary, and quinary classification models based on participants’ physiological signals and self-reported emotional responses through experiments in eight public spaces of five types. Furthermore, we verified the effectiveness of the model by inputting data collected from two other public spaces. Three observations were made based on the results. First, the highest accuracies of the binary and ternary classification models were 92.59% and 91.07%, respectively. After external validation, the highest accuracies were 80.90% and 65.30%, respectively, which satisfied the preliminary requirements for evaluating the quality of actual urban spaces. However, the quinary classification model could not satisfy the preliminary requirements. Second, the average accuracy of ensemble learning was 7.59% higher than that of single classifiers. Third, reducing the number of physiological signal features and applying the synthetic minority oversampling technique to solve unbalanced data improved the evaluation ability.

## Introduction

Affective computing has attracted significant interest in psychology, cognitive science, and computer science. Researchers have attempted to identify emotions and influencing factors through scientific and digital methods [1, 2]. Emotions can either be short- or long-term [3]. Short-term emotions are primarily related to stimuli and the corresponding response. Long-term emotions, on the other hand, are affected by more complex factors such as cultural background, politics, and time. Most researchers have focused on quantitatively revealing the relationships between people, stimuli, and emotions based on short-term emotions [4].

The emotional theories of James-Lange, Cannon-Bard, and Schachter-Singer, and the cognitive appraisal theory of Lazarus indicate that emotions are closely related to environmental stimuli and physiological responses [5–8]. Environmental stimuli emanate from events, weather, people, sounds, images, and scenery. Physiological responses include external responses (facial expression, language, and action) and internal physiological responses (the peripheral and central nervous systems) [9, 10]. In the fields of psychology, cognition, and computer science, researchers have used various typical or ordinary elicitations (objects), stimulated participants to elicit physiological responses, collected participants’ internal and external physiological data using instruments, and then built models of emotion recognition through data processing and feature extraction [10–12]. Most of these researchers collected data in the laboratory and selected typical pictures, videos, and sounds as stimuli. However, the actual applications occur in complex external environments that produce more data noise, and stimuli are frequently not typical images or sounds but daily and ordinary stimuli. In addition, although some researchers have used the same physiological signals as indicators, the features and classifiers were significantly different [12–25]. This results in a lack of comparability between related studies. Therefore, it is imperative to screen the main features and compare the classifiers to obtain a more reliable evaluation model. Furthermore, some researchers in urban design and geography have introduced emotion recognition methods and conducted related experiments in urban spaces [26–32]. However, they only selected a single type of space for data collection, such as a predefined route in the city center [27], shopping route in a city center [28], specific route around a city center [29], or predetermined route in a neighborhood [30], which makes data collection easy. However, this approach limits the scope of application of the model.

Generally, two approaches are used to evaluate the quality of urban public spaces: expert and user evaluations. The expert evaluation focuses on physical attributes, such as spatial form, color, material, and ecological conditions, and social attributes such as safety, function, and aesthetic quality. User evaluation, on the other hand, focuses on users’ perceptions, behaviors, and physiological data for a comprehensive evaluation. Because the evaluation indices of both approaches are significantly different, it is difficult to integrate them into one framework. Emotion quality evaluation belongs to the second method and is a quality evaluation method based on the user’s emotional experience. With the aid of contemporary physiological signal processing, feature extraction, and machine learning technology, emotional evaluation can be used to evaluate space quality based on the emotional response of many users by reducing the influence of individual factors. Ten public spaces of five types in Japan and China were selected for this study. We collected emotional signals emanating from the participants through real-world experiments and applied ensemble learning to build emotional classification models and spatial emotional quality evaluation processes. The proposed method is suitable for multiple types of urban public spaces and is easy to operate. The final evaluation results support spatial design and urban renewal decisions.

## Related research

Urban public spaces are a combination of the physical and social environments. The former includes artificial facilities, such as buildings and roads, and natural elements, such as microclimates, vegetation, and water. The combination and attributes of these elements in space can affect the environmental quality. The social environment includes security, function, aesthetic quality, and business conditions, which are partly related to the user’s experience and perception [28–30]. Therefore, it is difficult to determine the weight of each factor when evaluating the environmental quality as a whole through physical and social environments, particularly for different types of public spaces, because researchers assign different weights. Thus, it is difficult to apply a spatial quality evaluation system to new spaces.

Emotion is a comprehensive human response to environmental stimuli. As an evaluation index, emotion can prevent the problem related to weighting the evaluation factors. Although psychologists have not developed a widely accepted cognitive model for evaluating the quality of emotion, which is a black-box process, they generally consider two main processes when a person receives an external stimulus. The first process is called low-class evaluation, which is a relatively automatic evaluation of the initial cognitive and emotional responses to the stimulus. The second process, called higher-class evaluation, involves more explicit recognition and evaluation of the stimuli [7, 33]. Lazarus argued that cognitive activity precedes emotions, and emotions affect subsequent perception activities [8, 33]. Overall, scholars generally consider this process as an interaction between cognition and emotion; cognitive evaluation can elicit emotional responses that influence new cognition and judgment [8, 33, 34].

However, although users’ emotions are indicators of the quality of the public space, emotions are often influenced by subjective intentions. Thus, it is difficult to obtain accurate emotional data and important to determine the appropriate methods for measuring emotions. For this purpose, researchers have proposed two methods of emotional measurement: subjective and objective. The tools for measuring emotions subjectively include the self-assessment manikin (SAM), mood adjective scales, and positive and negative emotion scales. Although it is easy to obtain emotional responses using these tools, they are prone to subjective influences. The tools for measuring emotions objectively include physiological measurements, facial motion coding systems, and text analysis measurement methods [35–40]. This method is more advantageous because it prevents the subjective and deliberate influences of the participants. However, owing to technical limitations, the results of observable measurements cannot accurately reflect actual emotions. Therefore, most researchers combine subjective and objective measurement methods to reduce the data noise.

Over the past two decades, researchers in computer science, psychology, cognition, and physiology have used different methods to study emotion recognition. These researchers built various emotion recognition models by acquiring human physiological signals and extracting signal features [12, 13, 21, 22, 24, 41–46]. Typically, two types of emotional stimuli are selected. The first is virtual objects, such as pictures, videos, and music, and the other is actual environments, such as an in-car environment, building environment, street, and park. Virtual objects often include emotion labels. They are strong stimuli that are independent of the participants’ emotional responses [46, 47]. Actual environments are often weak stimuli without emotion labels and depend on the participant’s emotional response. Therefore, in contrast to using space photos or street view pictures as stimuli, experiments in actual three-dimensional space can yield emotional responses and physiological data that are more reflective of the actual scenario. Table 1 shows the related studies on emotion recognition using physiological sensors in urban spaces over the past decade.

**Table 1:** Related studies on emotion recognition using physiological sensors in urban spaces over the decade.

Reference	Sites	Number of Subjects	Signals	Number of Features	Emotions	Classifier	Results
Olsen and Torresen, [47]	a natural environment of daily life	10	accelerometer data	8 features	valence, arousal (3 classes)	SVM	arousal 75%, Valence 50.9%
Kalimeri and Saitis, [46]	a selected route in a campus	9	EEG, EDA	182 EEG features, 6 EDA features	5 predefined categories	RF	79.30%
Kanjo et al., [28]	a shopping route in a city center	40	EDA, HR, and body temperature	21 features	emotions	SVM, RF, KNN, and NB	86%
Birenboim et al., [27]	a predefined route in the city center	15	EDA, HR, and HRV	5 EDA and 3 HRV features	stress level	N/A	N/A
Kanjo et al., [29]	a specific route around a city center	40	HR, SGR, and body temperature	None	Emotions	CNN-LSTM	95%
Ojha et al., [30]	a predetermined route in a neighborhood	30	EDA	9 features	valence and arousal	REP-Tree, MLP, and SVM	87% (binary), 80% (multi-class)

Note: The full name of the abbreviation in the table: EEG: electroencephalograms; EDA: electrodermal activity; HR: heart rate; HRV: heart rate variability; SGR: specific growth rate.

Emotion recognition based on physiological signals includes seven steps: 1) selecting physiological signal feedback instruments and related equipment, 2) selecting emotional stimuli, 3) conducting experiments and collecting physiological signals, 4) extracting and reducing signal features, 5) fusing data, 6) selecting classifiers, and 7) verifying models. Among the related studies shown in Table 1, six researchers selected a single campus or space in a city center as a stimulus, and five researchers collected more than two physiological signals. All the researchers mainly used single-classification support vector machine (SVM), k-nearest neighbors (KNN), naïve Bayes (NB), convolutional neural network, long short-term memory (CNN-LSTM), multilayer perceptron (MLP), and ensemble classifier random forest (RF), and finally developed binary, ternary, and quinary emotional classification models.

### Physiological signal acquisition

The physiological signals related to emotions include external physiological responses and physiological signals. Owing to variations in cultures and habits, external physiological responses are diverse and may be subject to self-control. However, internal physiological signals are almost impossible to control. Therefore, they can be used as signs of emotional response [13, 17, 18, 24, 25, 34–40, 48–50]. The main physiological signals include EDA, electrocardiograms (ECG), electromyograms (EMG), EEG, and HRV. Most studies have indicated that using multiple physiological signals for emotion recognition is more effective than using a single physiological signal [15, 21–23, 46, 51–53].

### Extracting and reducing signal features

The features of physiological signals include time and frequency domains and nonlinear features. The number extracted by different researchers varies significantly because of the complexity of the features. The six researchers listed in Table 1 extracted 8 to 188 features, which led to different results. To date, the degree of correlation between features and emotions is inconclusive. Researchers frequently used principal component analysis (PCA) and factor analysis to reduce the number of features [52–58].

### Selecting classifiers

The selection of classifiers has a significant impact on recognition accuracy. Common classifiers suitable for emotion recognition include logistic regression (LR), SVM, decision trees (DT), artificial neural networks (ANN), and ensemble models such as RF [28–30, 59–62]. In addition to the selection of the classifier, the number of target variables had a more significant impact on the accuracy of recognition. Generally, the number of target variables was inversely proportional to the accuracy. Although the number of target variables input to the classifier ranged between two and five in related studies, but the accuracy was not significantly different [13, 25, 29, 30, 47, 48]. Meanwhile, there is no comparability between research results [28, 30, 53, 60, 62], and the accuracy of emotion recognition was considerably different.

## Methods

**Fig 1:**
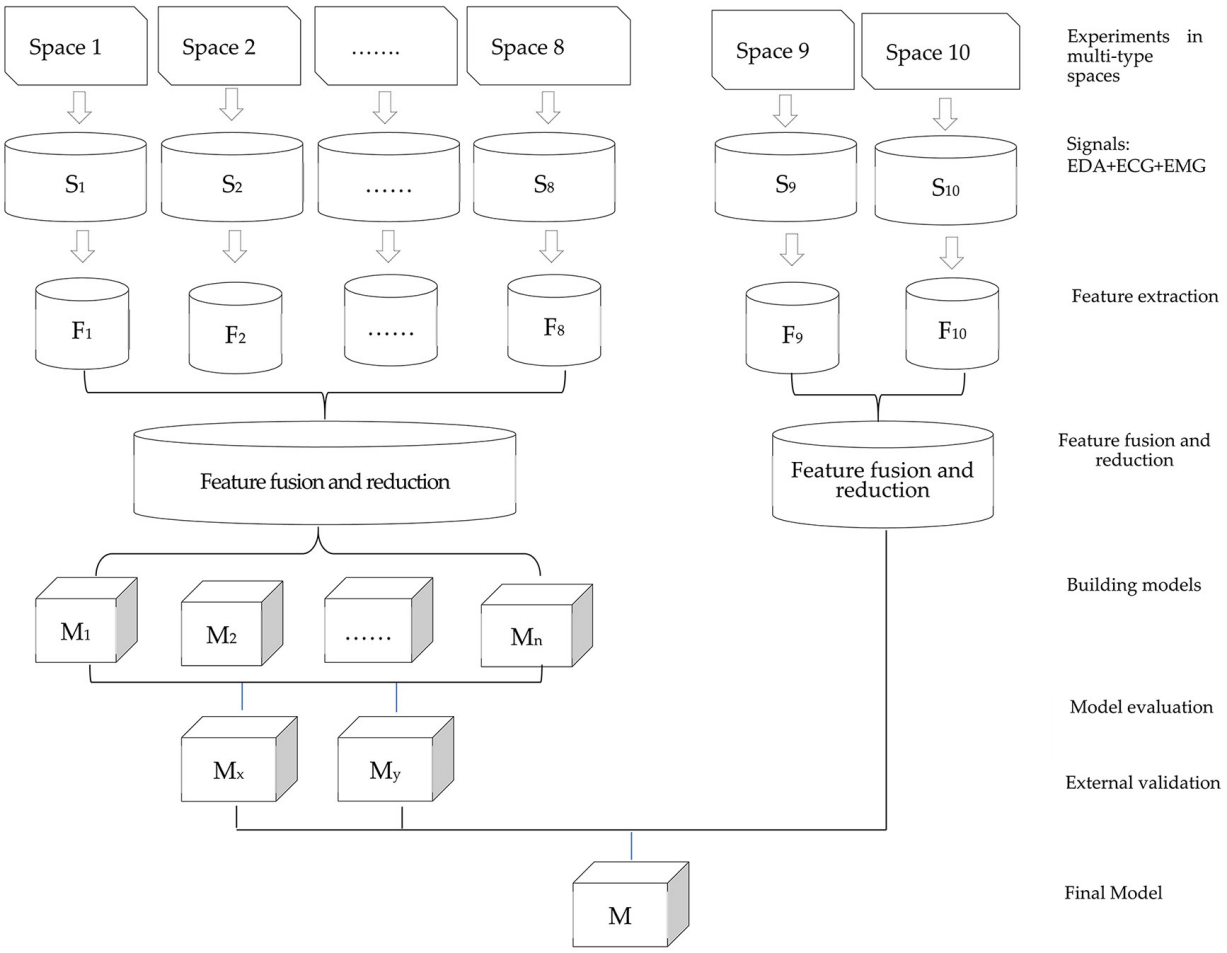
Flowchart of the experiment and data analysis process.

Typical urban spaces were selected as the stimuli to elicit the participants’ physiological and emotional signals. We built emotion recognition models using signal processing, feature extraction, and reduction. Fig 1 shows a flowchart of the study. In this process, we attempted to optimize the method of spatial emotion recognition and applied the proposed model to the public space of another city to further verify its effectiveness.

According to the provisions of Article 5, Paragraph 1 of the Regulations on the Conduct of Research Involving Human Subjects of the Japan Advanced Institute of Science and Technology (JAIST), we submitted a human body research plan to the Research Ethics Committee of JAIST and obtained research permission before the experiments. The research process followed the principles of the Declaration of Helsinki. The individual in this manuscript has given written informed consent (as outlined in PLOS consent form) to publish these case details.

### Data collection of urban public spaces

**Fig 2:**
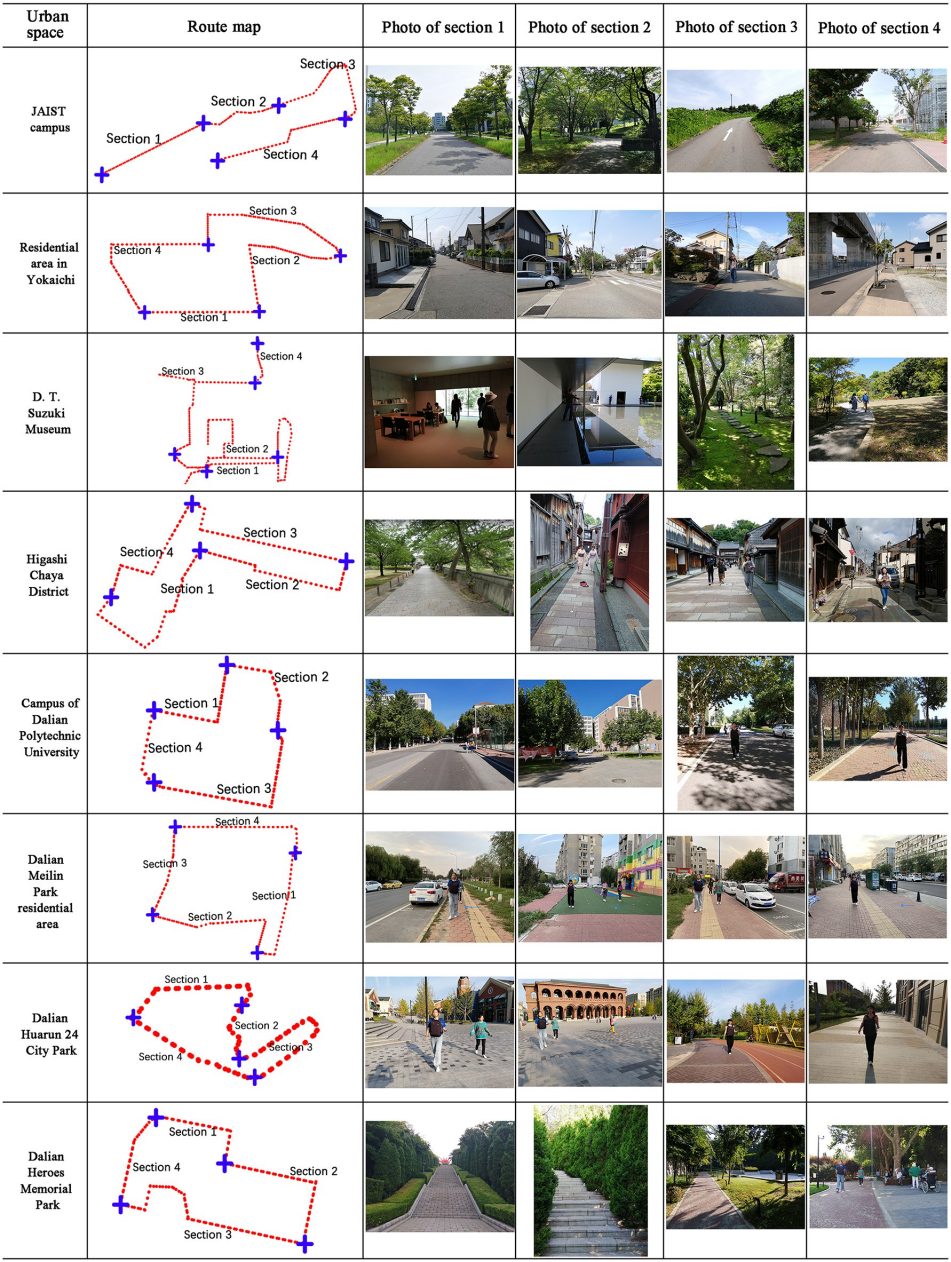
Route maps and photos of each section in the eight spaces; their data were used for model training and testing.

**Fig 3:**
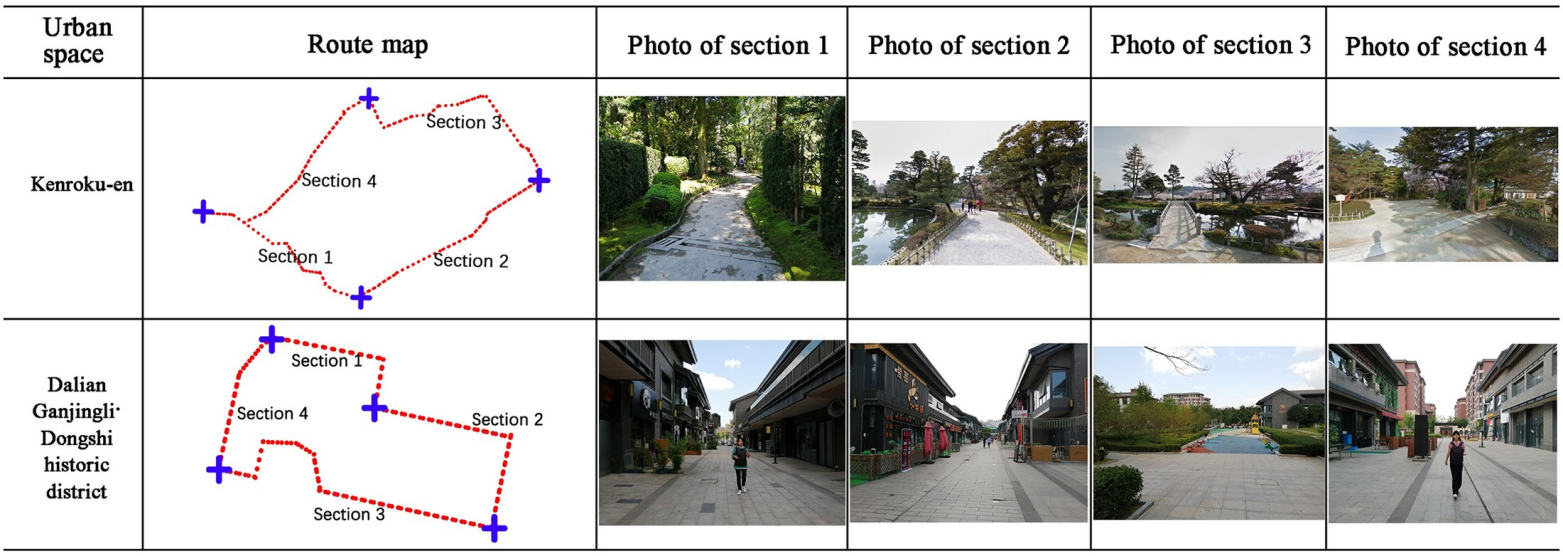
Route maps and photos of each section in the two spaces; their data were used for model external validation.

We collected data from 10 public spaces of five types: five in Nomi City, Kanazawa City, Japan, and five in Dalian City, China. The five types of spaces were campus public spaces, residential areas, park spaces, memorial spaces, and historical pedestrian street spaces. In each space, we selected a linear space with a length of approximately 300–1000 m as the experimental route and divided each route into four sections with different spatial characteristics (function and structure), for a total of 10 × 4 = 40 sections. Additionally, we divided these 10 spaces into the ratio of 8:2, used the data from eight spaces for model training and testing, and the data from the other two spaces for the external validation of the built model. Figs 2 and 3 show the route maps and photos of each section. The location, function, sections, and length of the selected spaces and experimental routes are listed in Tables 2 and 3. We used the data from the spaces in Fig 2 and Table 2 to train and test the models and those in Fig 3 and Table 3 to verify the model performance through external validation.

**Table 2:** Basic information on the eight spaces and their data were used for model training and testing.

Sites	Latitude and longitude	City	Function	Total length(m)	Section 1 (m)	Section 2 (m)	Section 3 (m)	Section 4 (m)
JAIST campus	36.4444, 136.5924	Nomi city (Japan)	Campus	697.5	180.4	123.8	192.3	201
Residential area in Yokaichi	36.5463, 136.6088	Kanazawa city (Japan)	Residential area	1067.9	188.1	280.6	299.3	299.9
D. T. Suzuki Museum	36.5578, 136.6608	Kanazawa city (Japan)	Monument	364.7	77.3	79.1	164.7	43.6
Higashi Chaya District	36.5720, 136.6668	Kanazawa city (Japan)	Pedestrian street	649.4	100.4	241.6	204.6	102.8
DLPU campus	38.9713, 121.5278	Dalian City (China)	Campus	651	161	136	251	103
Meilin Park residential area	38.9734, 121.5177	Dalian city (China)	Residential area	945	285	262	174	224
Huarun 24 City Park	38.9755, 121.5365	Dalian city (China)	City park	719	114	86	320	199
Dalian Heroes Memorial Park	38.9007, 121.6234	Dalian city (China)	Memorial site	788	247	95	294	152

**Table 3:** Basic information on the two spaces and their data were used for model external validation.

Sites	Latitude and longitude	City	Function	Total length(m)	Section 1 (m)	Section 2 (m)	Section 3 (m)	Section 4 (m)
Kenroku-en	36.5622, 136.6626	Kanazawa city (Japan)	Japanese garden	668.8	208.4	116.9	173.2	170.3
Dalian Ganjingli · Dongshi historic district	38.9508, 121.5350	Dalian city (China)	Pedestrian street	621.0	149.0	144.0	206.0	122.0

A total of 20 students (7 men and 13 women; average age, 28.6. Fourteen of them were aged 20–29, four aged 30–39, and two 40–49) participated in the experiment. There were nine participants in the experiments in Nomi City and Kanazawa City, Japan and 11 participated in the experiment in Dalian City, China.

**Fig 4:**
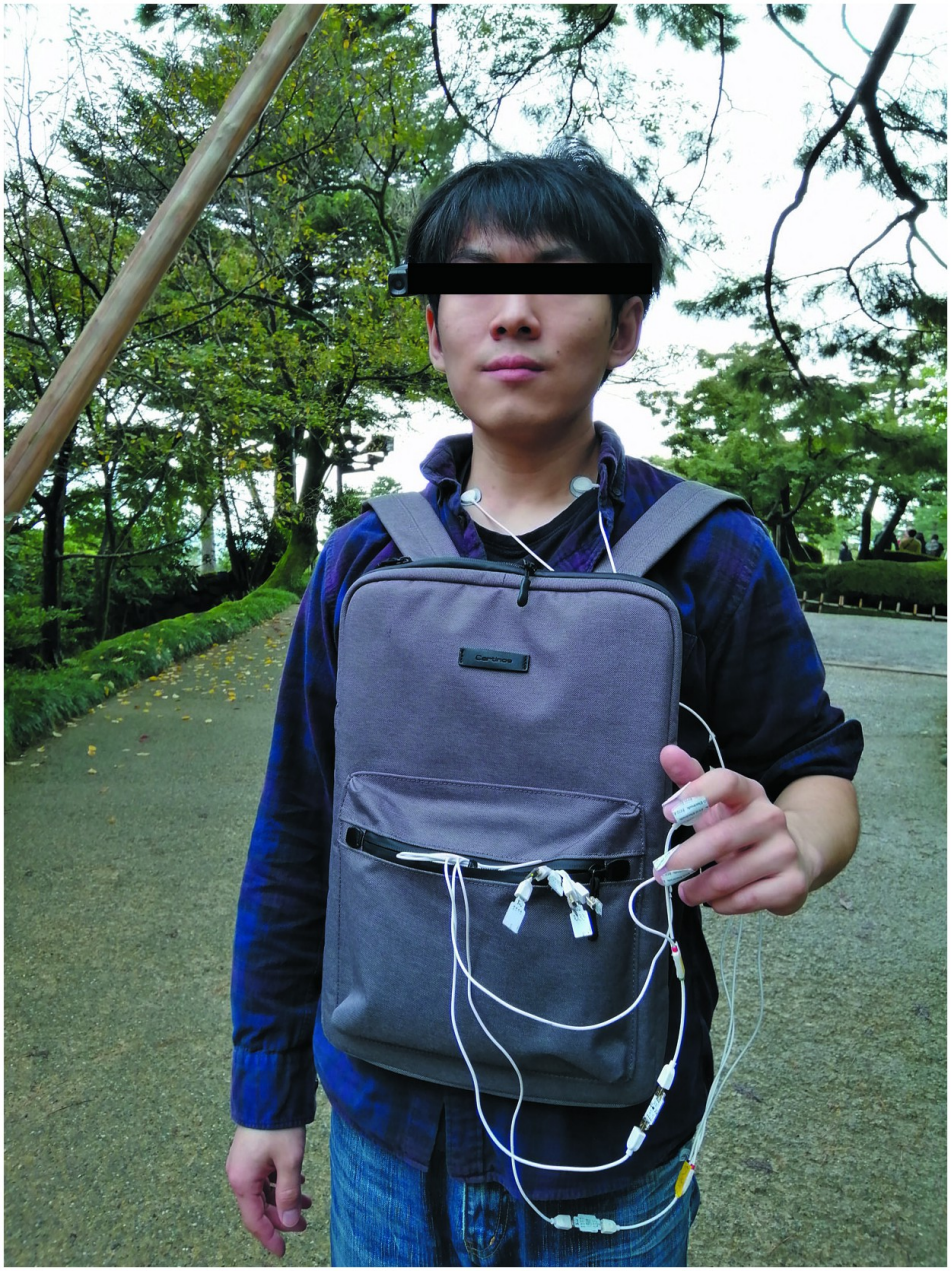
The participants wore a physiological signal feedback device (BITalino (r)evolution Plugged kit), carried a GPS device (Nav-u NV-U73T, Sony) and a laptop computer as they walked through the selected space route at a natural pace and constant speed. They could turn their heads to look at their surroundings. The computer automatically recorded the EDA, ECG, and EMG. The position where the electrodes of the physiological signal feedback instrument were pasted on the body: the two EDA electrodes were fixed on the first phalanx of the index and middle fingers; the ECG electrodes were fixed at the carotid arteries on both sides of the neck; the EMG electrodes were fixed on the inner side of the forearm of the arm.

**Fig 5:**
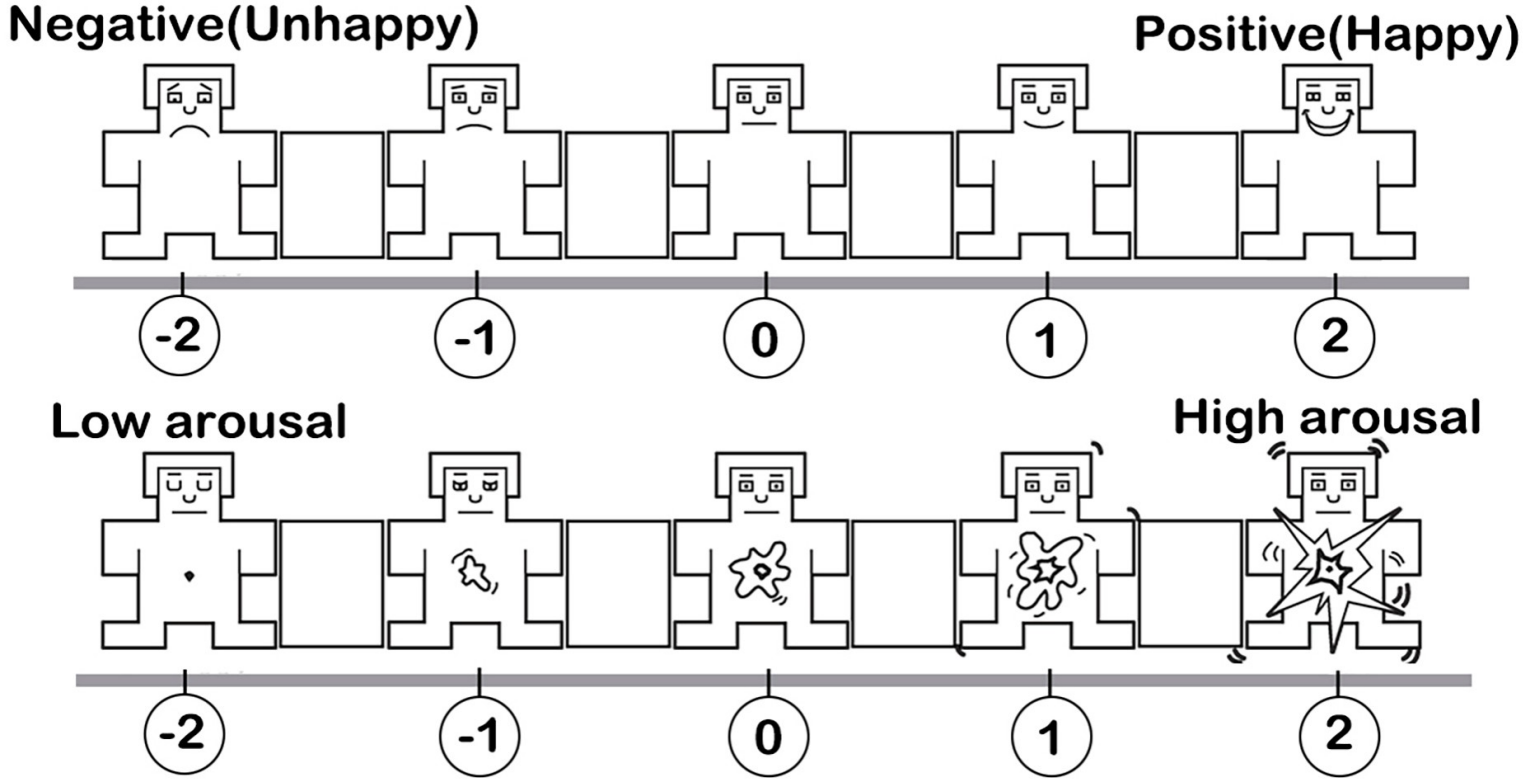
Self-assessment manikin (SAM) scale [63].

Except for the two campuses, none of the participants visited any of the sites before the experiment. Prior to the experiment, the aims and experiment content were explained to each participant. All the participants signed a formal consent form. During the experiment, the participants wore a Bitalino portable physiological signal feedback instrument (BITalino (r)evolution Plugged kit, PLUX Wireless Biosignals Ltd., Portugal), carried a GPS device (Nav-u NV-U73T, Sony), and walked through the five spaces. The physiological signal feedback instrument collected the participants’ EDA, ECG, and EMG, which were stored on a laptop in the backpack. The GPS recorded the participants’ location information simultaneously (Fig 4). Each participant filled out the SAM immediately after walking through each space (Fig 5).

### Data processing and analysis

#### Emotional valence and arousal

The SAM is a straightforward and universal tool that can track individual responses to emotional stimuli in various environments and rapidly evaluate emotional responses. During the experiment, affective information were obtained from the participants’ responses to the SAM questionnaire. Because it is an odd options of emotion measurement tools, we could obtain three, five, and seven emotion levels. To build a binary classification model, we deleted the samples whose emotional valence was zero and considered emotions whose valence was -2 and -1 as negative emotions and marked them as“-1”; those whose valence were one and two were positive emotions and marked as “1.”

In addition, the statistical results of the SAM scale indicated that, compared to the meaning of emotional valence (positive or negative), emotional arousal was less understood by the participants, who found it difficult to distinguish between emotional arousal and psychological stress. Furthermore, some participants stated that they would experience psychological stress caused by individual differences as they walked through the public space while wearing instruments and stress can interfere with emotional arousal. Therefore, we did not use emotional arousal. Rather, we used only emotional valence as the target variable to build the valence classification model (S1 Table).

#### Physiological signal preprocessing

Noise reduction was necessary because the physiological signals collected in public urban spaces contained more noise. The interference in the ECG signal primarily results from power frequency interference, electrode contact noise, electromyographic noise, and breathing. Therefore, we used a Butterworth filter to low-pass filter the ECG signals and applied a zero-phase-shift filter to correct the baseline drift. The denoising of the EDA signal included smoothing, denoising, and filtering using a second-order Butterworth filter with a cut-off frequency of 0.3 Hz. The EMG signal is a waveform diagram of the action potential generated by muscle contraction. Because of the influence of the participants’ walking movements, we applied the Blackman window algorithm to the EMG signal for high- and low-pass filtering (5–50 Hz).

#### Feature extraction and reduction

Based on the GPS positioning, we divided each participant’s EDA, ECG, and EMG signals in each space into four segments; thus, each signal had 400 samples. As 22 samples were incomplete, 378 were valid.

To ascertain the number and effectiveness of the features, we applied different software packages to extract features from the EDA, ECG, and EMG signals. First, we used AcqKnowledge (ver. 4.2) [64] to analyze the EDA signal and obtain seven time-domain and five nonlinear features. After the Fourier transform, we obtained four frequency-domain features (S1 Fig). We then used Kubios HRV Premium software (ver. 3.4.3) [65] to extract the features of the ECG signal and obtained 17 time-domain features, 16 frequency-domain features, and 33 nonlinear features (S2 Fig). We used the plug-in EMG Toolbar V5.30 [66] of the software Origin 2019 [67] to extract the features of the EMG signals and obtained five time-domain features and two frequency-domain features (S3 Fig). We obtained a total of 68 signal features (Table 4 and S2 Table).

**Table 4:** Sixty-eight extracted signal features (all) and 50 reduced features (Italics are deleted features).

EDA signal features (8/16)	ECG signal features (36/45)		EMG signal features (6/7)
Max	Mean RR	HF	Integ.
Min	SDNN	Total power	RMS
Mean	Mean HR	LF/HF ratio	Mean
Stddev	SD HR	SD1	SD
Median	Min HR	SD2	F. mean
Mutual Information	Max HR	SD2/SD1 ratio	F. med.
Median F	RMSSD	Approximate entropy	
Kurtosis	NNxx	Sample entropy	*Max*
	pNNxx	alpha 1	
*Min F*	RR tri index	Mean line length	
*Skew (1)*	TINN	Recurrence rate	
*Skew*	DC	Determinism	
*Kurtosis (1)*	DCmod	Shannon entropy	
*Capacity dimension*	AC		
*Correlation dimension*	ACmod	*VLF (Hz)*	
*Information dimension*	LF	*LF (Hz)*	
*Lyapunov exponent*	HF	*HF (Hz)*	
	VLF	*VLF* (*ms*^2^)	
	LF	*VLF (%)*	
	HF	*EDR*	
	LF	*alpha 2*	
	HF	*Correlation dimension*	
	LF	*Max line length*	

We then used SPSS (IBM SPSS Statistics 24) to perform PCA on 68 signal features. The results indicated that the significance of the Bartley sphere test was P<0.01, KMO = 0.795, PCA was effective, and the value of extracted eigenvalues was greater than 1 (cumulative % = 85.78%) in the components. After calculating and comparing the weight of each feature, we selected 50 features (shown in bold text in Table 4) that were highly correlated with emotions (S3 Table).

### Building models and evaluation methods

We obtained a total of 10 datasets, including valence and feature data from 10 spaces. We used eight of these Table 2 for model training and testing (S1 Text). The other two datasets Table 3 were used as new data to verify the classification capability of the proposed model. We then used SPSS Modeler18.1 to establish the training and validation models of binary, ternary, and quinary classifications.

#### Unbalanced data and synthetic minority oversampling technique (SMOTE)

**Fig 6:**
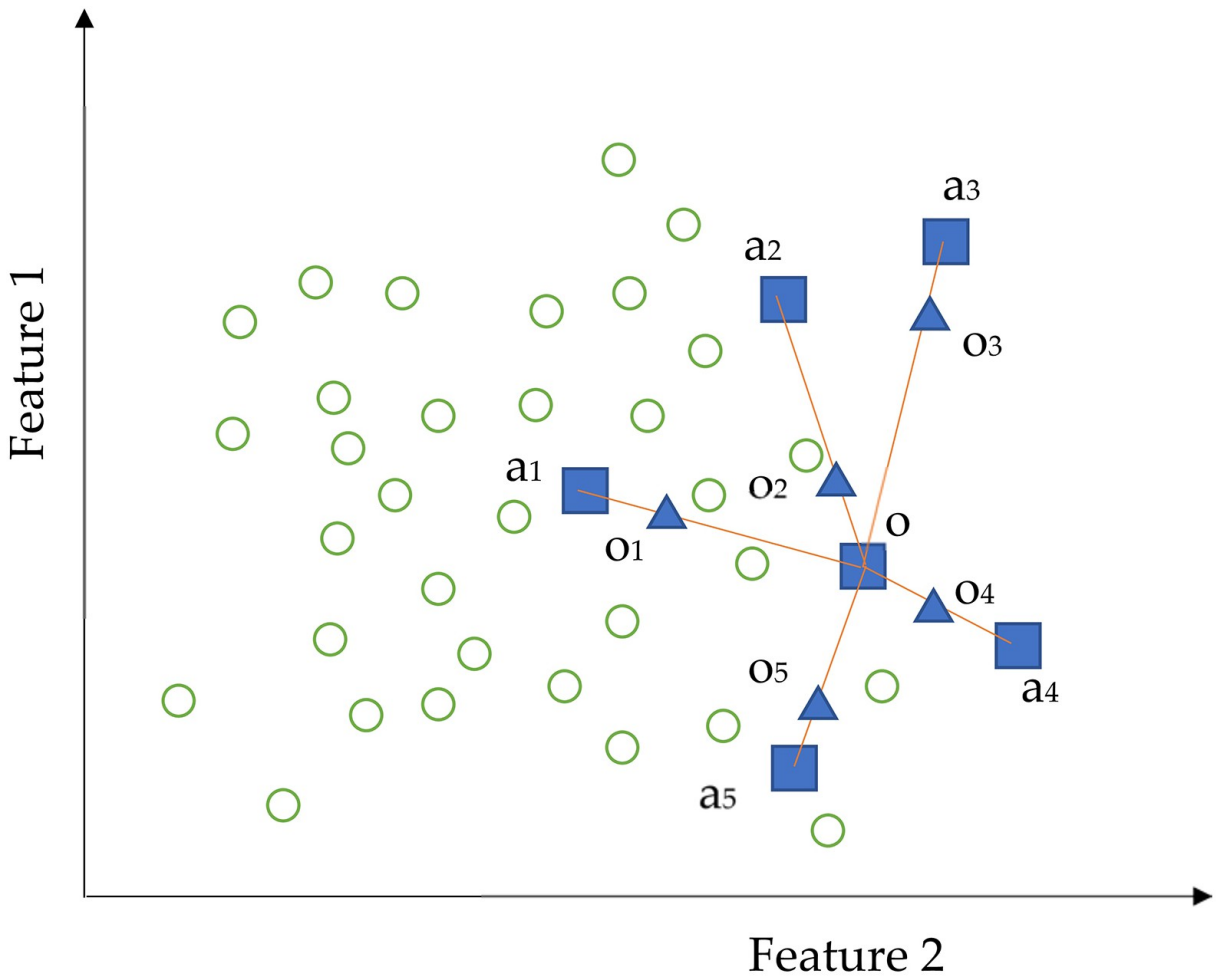
SMOTE algorithm: The blue square and green circle represent the minority and majority classes, respectively. The KNN of point O in the minority set was obtained by calculating the Euclidean distance between O and each sample in the set. Based on the k (k = 5), the algorithm connected the k (k = 5) minority points (a1, a2, a3, a4, a5) around O, and finally inserted new synthetic points (O1, O2, O3, O4, O5) on the line of the two points, until the number of all the minority types and insertion points was balanced with the number of majority types.

The public space built in a city is primarily a place for citizens’ daily leisure and entertainment; thus, the emotions elicited by the space stimulation are primarily positive or calm. Therefore, in the collected data, we observed that the samples of “valence = -2” and “valence = -1” in the dataset were significantly less than other samples, which resulted in poor recognition of negative emotions in the training model. Therefore, we introduced the SMOTE to solve the problem of unbalanced data. Class imbalance refers to an unbalanced distribution of classes in the training set. The proportion of the minority class is equal to or less than 10% of the dataset. When the data is unbalanced, the minority classes do not provide sufficient “information”, and the model cannot accurately predict the minority classes. SMOTE is an improved oversampling method [68] that randomly selects an example from a minority group and determines its k-nearest neighbors (KNN) (k = 5 in this example). Subsequently, the algorithm randomly selects a neighborhood in the feature space, as well as a point between the two samples as a new sample, repeats the above steps, and finally achieves a balance between the majority and minority samples (Fig 6).

#### Single and ensemble classifiers

In related research, the classifiers used were single classifiers, including LR, SVM, DT 5.0, ANN, and RF ensemble classifier [28–30, 59–62]. We used three single classifiers and three ensemble classifiers for the model training. The single classifiers were LR, DT, and ANN, and the three ensemble classifiers were DT C5.0 (boosting), RF (bagging), and the neural network (boosting).

Ensemble learning achieves a better predictive performance by combining predictions from multiple models. The three main classes of ensemble learning methods are bagging, stacking, and boosting methods. Among these, bagging and boosting are used more often than stacking. Bootstrap aggregation (bagging) is an ensemble learning method that achieves a diverse group of ensemble members by varying the training data. Boosting is a machine learning algorithm that can be used to reduce deviations in supervised learning. Boosting learns a series of weak classifiers and combines them into a robust classifier. To avoid overfitting and achieve a high classification accuracy, we compared the performance indices of the models, and finally selected the models with solid generalization ability.

#### Selection of performance indicators of the model

The confusion matrix, also known as the error matrix, is a standard format for accuracy evaluation. It can be used to calculate the performance indices of the classification model: accuracy, recall, and F1-score. The calculation method for each index is as follows.

Accuracy=(TP+TN)/(TP+TN+FP+FN)

$$\begin{array}{c} Recall(R)=TP/(TP+FN) \end{array}$$

$$\begin{array}{c} F1-Score=2P*R/(P+R) \end{array}$$

Note:

*TP = No. of true positives among total predictions*;

*FP = No. of false positives among total predictions*;

*FN = No. of false negatives among total predictions*;

*TN = No. of true negatives among total predictions*.

In addition to the above three indices, we also selected the area under the curve (AUC) and the Gini coefficient as the performance indices of the binary classification model. The AUC is a popular measure of the degree or measure of separability. This indicates the extent to which the model is capable of distinguishing between the two classes. The value range of the AUC is between 0.5 and 1. An AUC of 0.5 indicates the worst performance. The closer the AUC is to 1.0, the better the performance of the model. The Gini coefficient compares the Lorenz curve of a ranked empirical distribution to the line of perfect equality. It measures the degree of concentration (inequality) of a variable within the distribution of its elements. It is calculated as follows:

$$\begin{array}{c} Gini\;coefficient=area\;a/(area\;a+area\;B)=twice\;the\;area\;A \end{array}$$

For the indices of the ternary and quinary class classification models, we also selected Cohen’s kappa coefficient to test the consistency of the classification results. Cohen’s kappa is a statistical coefficient that represents the degree of accuracy and reliability of the classification. It measures the agreement between two raters who classify items into mutually exclusive categories [69]. The kappa value is always less than or equal to one, indicating less-than-perfect or perfect agreement, respectively. The Cohen’s kappa coefficient was calculated as follows:

$$\begin{array}{c} k=\left(p_{o}-p_{e}\right)/\left(1-p_{e}\right) \end{array}$$

where *p*_*o*_ is the relative observed agreement among raters, and *p*_*e*_ is the hypothetical probability of chance agreement.

## Results

### The effect of feature reduction on the models

The PCA algorithm was used to reduce the extracted 68 features to 50. However, although the PCA algorithm reduced the dimension of the independent variables, the significance of these independent variables to the target variable was not clear. To verify whether the reduction in the number of features had a positive effect on valence classification, we used 68 and 50 signal features to build binary and ternary classification models, respectively (RF (bagging) and ANN (boosting) as classifiers). Table 5 presents the results of the model performance before and after feature reduction.

**Table 5:** Comparison of the performance of the models based on 68 features and 50 features.

Class	Classifier	68 features					50 features				
		Accuracy	Recall	F1-Score	AUC	Kappa	Accuracy	Recall	F1-Score	AUC	Kappa
Binary	RF (bagging)	75.90%	0.78	0.755	0.886		83.30%	0.833	0.816	0.951	
	ANN (boosting)	85.20%	0.855	0.855	0.879		92.60%	0.929	0.929	0.962	
Ternary	RF (bagging)	87.10%	0.87	0.87		0.804	91.10%	0.917	0.91		0.866
	ANN (boosting)	83.60%	0.823	0.827		0.75	90.20%	0.907	0.9		0.852

### Classification results and performance comparison

#### Binary classification

We divided the eight datasets used for the training and testing models into two parts, in the ratio of 8:2, which were randomly selected as the training and test sets, respectively (S4 Table) The values of the target variable for binary classification were “-1, 1,” and 50 signal features as the independent variables. The model performance results are presented in Table 6 and S4 Fig.

**Table 6:** Performance comparison of binary classification models with different classifiers.

Classifiers	Recall	F1-Score	AUC	Gini	Accuracy
LR	0.679	0.679	0.718	0.436	74.29%
DT C5.0	0.571	0.571	0.642	0.285	65.71%
ANN	0.964	0.931	0.917	0.833	94.29%
DT C5.0 (boosting)	0.75	0.706	0.888	0.777	72.22%
RF (bagging)	0.833	0.816	0.932	0.865	83.33%
ANN (boosting)	0.929	0.929	0.971	0.942	92.59%

The results of binary classification indicated that the recognition accuracies of the models based on the ANN and ANN (boosting) were higher than 90%, and they had better classification performance. These results also indicate that the two models was effective for evaluating the affective quality evaluation of urban public spaces.

#### Ternary classification

**Fig 7:**
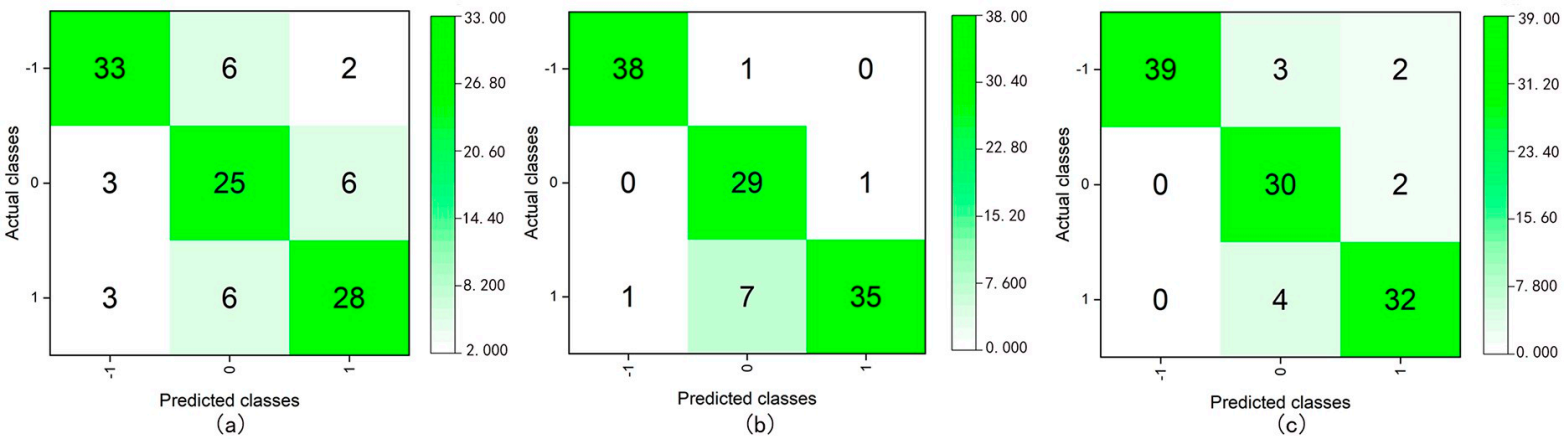
Confusion matrices of the ternary class classification using DT C5.0 (boosting) (a), RF (bagging) (b), and ANN (boosting) (c).

The value of the target variable for ternary classification were “-1, 0, and 1”, and all the valid sample data were used in model training or testing. The sample data were divided into training and test sets at a ratio of 8:2, and SMOTE was used for data oversampling (S5 Fig). After testing the models, we obtained the classification accuracy and average of each class of model performance index, as presented in Table 7 and Fig 7.

**Table 7:** Performance comparison of ternary classification models with different classifiers.

Classifiers	Recall	F1-Score	Kappa	Accuracy
LR	0.62	0.623	0.432	62.20%
DT C5.0	0.89	0.877	0.808	87.40%
ANN	0.77	0.77	0.642	76.38%
DT C5.0 (boosting)	0.767	0.763	0.651	79.17%
RF (bagging)	0.917	0.91	0.866	91.07%
ANN (boosting)	0.907	0.9	0.852	90.18%

The performance indices of each class classification in the ternary classification model are listed in Tables 8 and 9.

**Table 8:** Performance indexes of each class classification in the ternary classification model with three single classifiers.

Valence	LR			DT C5.0			ANN		
	Recall	F1-Score	Accuracy	Recall	F1-Score	Accuracy	Recall	F1-Score	Accuracy
-1	0.62	0.68	80.31%	0.97	0.88	0.94	0.87	0.9	94.49%
0	0.59	0.56	71.65%	0.87	0.89	0.92	0.78	0.67	80.31%
1	0.65	0.63	72.44%	0.83	0.86	0.89	0.68	0.74	77.95%

**Table 9:** Performance indexes of each class classification in the ternary classification model with ensemble learning.

Valence	DT C5.0 (boosting)			RF (bagging)			ANN (boosting)		
	Recall	F1-Score	Accuracy	Recall	F1-Score	Accuracy	Recall	F1-Score	Accuracy
-1	0.8	0.82	87.50%	0.97	0.97	98.21%	0.89	0.94	95.54%
0	0.74	0.7	81.25%	0.97	0.87	91.96%	0.94	0.87	91.96%
1	0.76	0.77	84.82%	0.81	0.89	91.96%	0.89	0.89	92.86%

From the results of the ternary classification, we observed that the models based on the ANN (boosting) and RF (bagging) had higher performance index values and their recognition accuracies were 91.07% and 90.18%, respectively. Moreover, the models exhibited better classification abilities for each class (Fig 7). The results indicated that both models could also effectively evaluate the affective quality of urban public spaces.

#### Quinary classification

**Fig 8:**
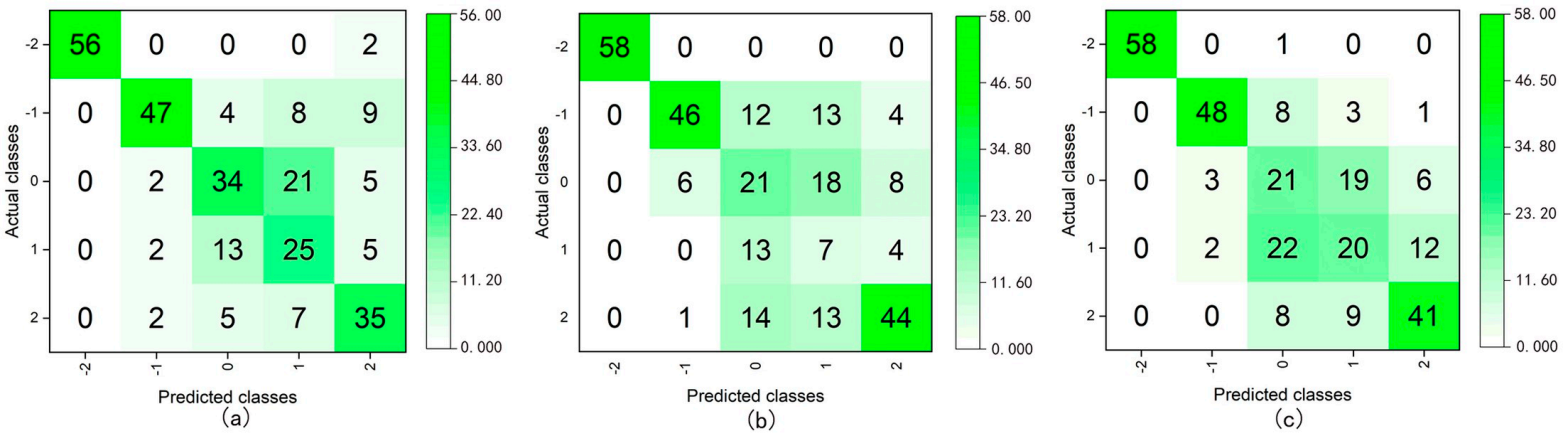
Confusion matrices of the quinary class classification using DT C5.0 (boosting) (a), RF (bagging) (b), and ANN (boosting) (c).

The value of the target variable for quinary classification was “-2, -1, 0, 1, 2”, and all the valid sample data were used to build the models. We divided the sample data into training and test sets according to a ratio of 8:2 and used SMOTE for data oversampling (S6 Fig). After testing the models, we obtained the following classification accuracy and average of Recall, F1-score, and Kappa for each class, which are presented in Table 10 and Fig 8.

**Table 10:** Performance comparison of quinary classification models with different classifiers.

Classifiers	Recall	F1-Score	Kappa	Accuracy
LR	0.562	0.552	0.531	57.43%
DT C5.0	0.594	0.576	0.561	60.32%
ANN	0.624	0.636	0.587	61.12%
DT C5.0 (boosting)	0.696	0.696	0.624	69.86%
RF (bagging)	0.656	0.658	0.584	66.67%
ANN (boosting)	0.582	0.59	0.529	62.41%

The performance indices of each class classification in the quinary classification model are listed in Tables 11 and 12.

**Table 11:** Performance indexes of each class classification in the quinary classification model with three single classifiers.

Valence	LR			DT C5.0			ANN		
	Recall	F1-Score	Accuracy	Recall	F1-Score	Accuracy	Recall	F1-Score	Accuracy
-2	0.51	0.55	56.61%	0.59	0.58	61.26%	0.52	0.56	60.16%
-1	0.77	0.77	78.20%	0.73	0.71	74.25%	0.74	0.75	76.35%
0	0.45	0.43	45.12%	0.46	0.5	55.77%	0.51	0.48	55.61%
1	0.51	0.5	50.12%	0.51	0.56	56.06%	0.55	0.54	57.56%
2	0.59	0.59	60.01%	0.61	0.69	70.56%	0.54	0.6	62.91%

**Table 12:** Performance indexes of each class classification in the quinary classification model with ensemble learning.

Valence	DT C5.0 (boosting)			RF (bagging)			ANN (boosting)		
	Recall	F1-Score	Accuracy	Recall	F1-Score	Accuracy	Recall	F1-Score	Accuracy
-2	0.97	0.98	99.29%	0.98	0.99	99.65%	1	1	100.00%
-1	0.69	0.78	90.43%	0.8	0.85	93.97%	0.61	0.72	87.23%
0	0.55	0.58	82.27%	0.43	0.39	76.24%	0.4	0.37	74.82%
1	0.56	0.47	80.14%	0.36	0.37	76.24%	0.29	0.19	78.37%
2	0.71	0.67	87.59%	0.71	0.69	87.23%	0.61	0.67	84.40%

The results of the quinary classification indicated that the model that incorporated DT C5.0 (boosting) had the best classification performance. However, its accuracy was only 69.86%, and the kappa coefficient was low, which demonstrated that the recognition performance of each class was very uneven, although some classes had 100% accuracy. Thus, in practice, these six models cannot satisfy the quinary classification of the affective quality of a space.

**Fig 9:**
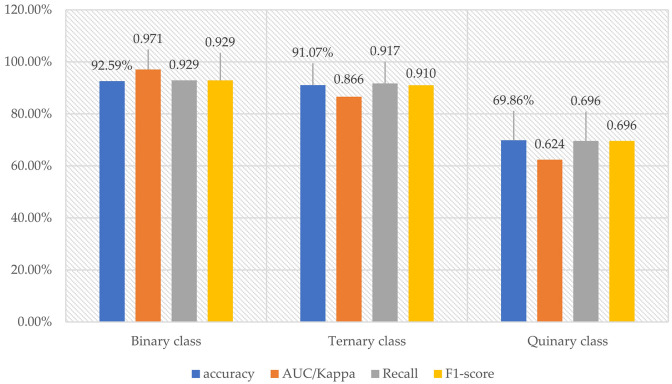
Comparison of accuracy and main performance indexes (binary: AUC; ternary and quinary: Kappa) of binary, ternary, and quinary classification models.

The comparison of the four indices of the binary, ternary, and quinary classification models with the best performance is shown in Fig 9. The results indicated that the classification ability declined sequentially, and that the quinary class classification had a significant decline. The binary and ternary class classification models were proven to be able to satisfy the practical requirements.

### External validation

**Fig 10:**
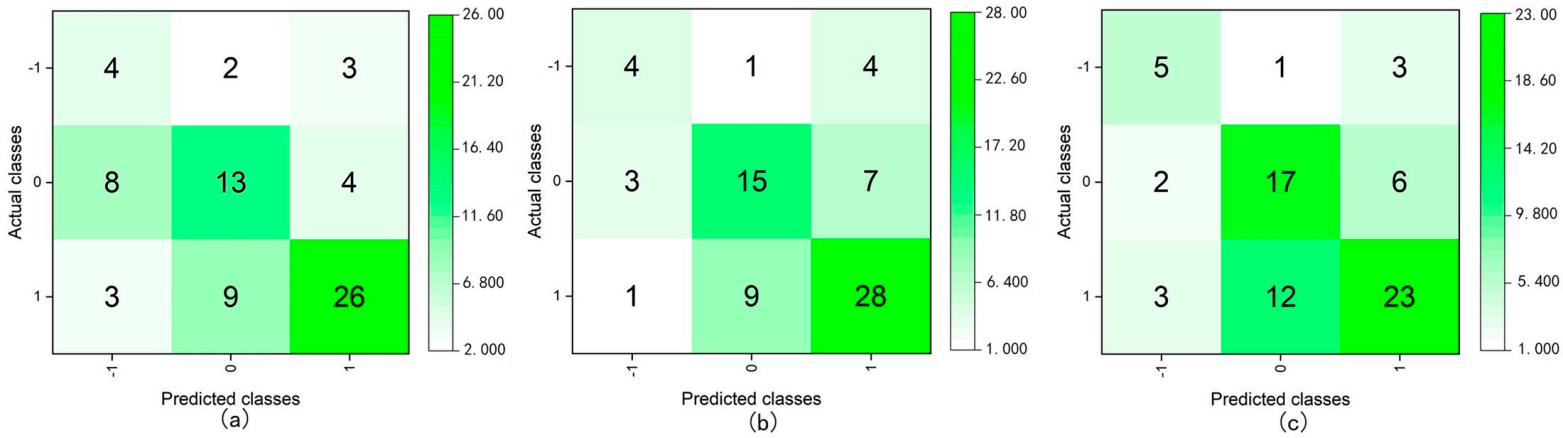
Confusion matrices of the ternary class classification for external validation using DT C5.0 (boosting) (a), RF (bagging) (b), and neural network (boosting) (c).

**Fig 11:**
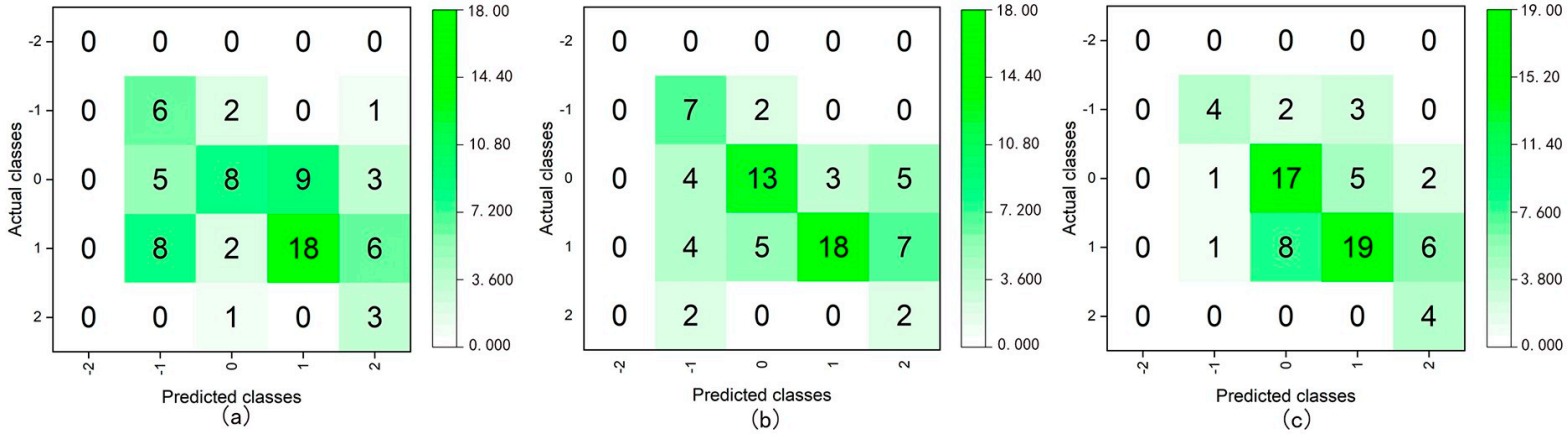
Confusion matrices of the quinary class classification for external validation using the DT C5.0 (boosting) (a), RF (bagging) (b), and ANN (boosting) (c).

In addition to internal testing, the performance of the models was subjected to external validation. We input the two previously selected spatial datasets (collected from Japan and China) into the built binary, ternary, and quinary classification models to verify the effectiveness of the model at predicting new spatial emotional quality (S7 Fig). The models output results for the two spaces. By comparing the output classification results with the raw valence values, we obtained the accuracy and confusion matrices of the classification, as shown in Table 13, Figs 10 and 11 (S5 Table).

**Table 13:** Classification accuracy of the emotional quality of the two new spaces using the proposed binary, ternary, and quinary-class classification models.

Models	Binary	Ternary	Quinary
LR	46.80%	44.40%	43.10%
DT C5.0	65.90%	53.30%	51.40%
ANN	61.70%	56.90%	55.60%
DT C5.0 (boosting)	65.90%	54.20%	47.20%
RF (Bagging)	78.70%	65.30%	55.60%
ANN (boosting)	80.90%	62.50%	61.10%

The results indicated that the highest accuracy of external validation in binary classification was 80.9%, whereas those of ternary and quinary types were 65.3% and 61.1%, respectively. Moreover, the accuracies of the ensemble classifiers were generally higher than those of the corresponding single classifiers. The confusion matrix of the ternary classification indicated that the classification results of samples whose valences were -1 were lower than those of the other classes. Because there was no sample whose valence was -2 in the new data, the quinary classification result was zero and the classification results of the samples whose valences were zero and one were more accurate than those of the others.

### Application process of the proposed model

The training model was designed for evaluating the quality of public spaces in practice. Furthermore, the external validation described in the previous section was aimed at not only the verification of the model, but also the application of the model in practice. These two steps verified the effectiveness of the model in practice.

**Fig 12:**
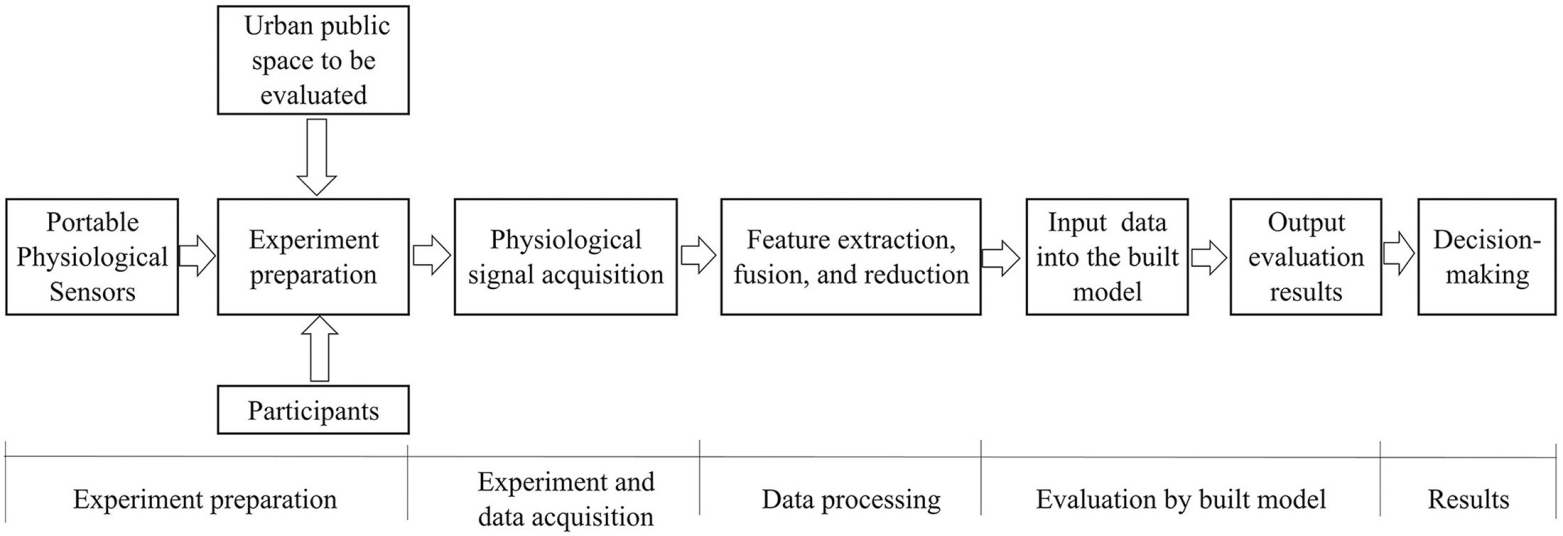
Emotional quality evaluation process of urban built public space.

Therefore, we attempted to develop a process for evaluating the affective quality of urban public spaces based on multiple physiological signals (Fig 12). The process entailed the following steps. First, we determined the experimental routes and divided them into several sections. Then, we invited the local community residents to participate and sign the consent form. In the data collection stage, we collected several physiological signals when the participants walked through these routes. After feature extraction, fusion, and reduction, the features were input into the classification model. According to the results, a space with a positive valence will maintain the status quo, whereas a space with a negative valence required renovation.

## Discussion

Models suitable for evaluating the affective quality of multitype public spaces were built and examined in this study. We not only improved the model’s performance through feature selection, SMOTE, and ensemble classifiers but also used external validation to verify the actual performance of the model.

The aims and methods of the proposed approach differed from those of extant approaches. First, to ensure the adaptability of the model, the scope of this study was multi-type spaces across countries. Second, we used three ensemble classifiers and compared their performances with those of single classifiers. In the past 10 years, ensemble classifiers have demonstrated strong classification performance. Compared with the models established using classifiers, such as SVM [28, 30, 47], KNN [28], BEP-tree [30], MLP [30], and RF [28, 46], in related studies, the ensemble classifiers used in this study exhibited a higher classification accuracy, 92.59% in binary class classification and 91.07% in ternary class classification, and supported by the higher performance indices. For the quinary classification, the highest accuracy in this study was 69.86%, which was lower than the 79% obtained by Kalimeri and Saitis [46]. We attributed this to the single-space experiments and similar emotional responses. The features of each part of the same space were generally not significantly different; thus, although high accuracy was achieved, the diversity of the spatial emotions and the adaptability of the model were reduced. Third, the proposed model was subjected to external validation to circumvent the limitations arising from sourcing the data of the training and validation sets from the same space. Thus, new data were inputted into the model and the results indicated that the performance of the model decreased significantly; specifically, for the multiclass classification model, the decline was between 5%-30%. Therefore, we confirmed that classification studies cannot be performed using only a unified dataset, and external verification is necessary.

As shown in Table 1, previous researchers extracted 8–188 features from physiological signals. This considerable difference in the number of features was owing to the difference in the number of physiological signals and the feature extraction method. Therefore, to ensure the comparability of the studies and facilitate their operation in practical applications, we selected three commonly used physiological signals, EDA, ECG, and EMG, and the PAC method, which is widely used to reduce the feature dimensions. As shown in Table 10, with the same classifier, the recognition accuracy of the model increased by 6.35% on average, after the number of features was reduced from 68 to 50; other indices improved as well. These results indicated that the PAC algorithm effectively eliminated data redundancy and noise and improved the classification ability of the model. However, obtaining a definite number of features remains a challenge and solving this problem requires scholarly consensus following extensive experiments.

Compared with positive emotions, relatively fewer spaces, unless they are undeveloped or under problematic management, elicit negative emotions. Thus, we had a situation where the data sample contained insufficient examples of negative emotions, occasionally, less than 1/10 of the positive emotion samples. The unbalanced samples resulted in inaccurate predictions. Generally, up-sampling and down-sampling the data or algorithm level can solve this problem; however, simply increasing the amount of data by duplication affects a model’s adaptability. On the other hand, directly reducing the sample size results in information loss. Oversampling techniques, such as SMOTE, increase the number of minority samples. Additionally, it has minimal effect on the information contained in the data, making it possible to obtain a model with better classification ability.

By calculating the average difference among the accuracies of the three ensemble classifiers and the three single classifiers in Tables 5, 6 and 8, we observed that the average accuracy of the ensemble classifiers was 7.59% higher than that of the single classifiers. A comparison of the Gini and Kappa coefficients yielded similar results, which indicated that these ensemble classifiers adapted better to the multi-noise data collected in urban public spaces. Moreover, the performance of the models with ANN (boosting) and RF (bagging) classifiers was better than that of the model with DT C5.0 (boosting). These results may be attributed to the greater data fault tolerance of neural network (boosting) and RF (bagging) in comparison to DT C5.0 (boosting). Users’ emotions are affected by a variety of spatial factors; therefore, the fault tolerance of the models was significant.

External validation is a method for validating the predictive ability of a model by entering a new dataset. Related studies have shown that good test results do not guarantee that a model will have good adaptability. The predictive ability of the model for new data is often lower than that of the test results [70–72]. Similar results were obtained in our study. The results of the external validation of the quinary classification were significantly worse than those of the test results. We attributed this to the use of different spatial data and participants, as well as the limited sample size of external verification. Quinary classification requires a larger sample size than binary and ternary classification. Meanwhile, as a comparison of Figs 7 and 9 reveals, the two classification results were almost the opposite. In the classification of the test set, the classification results of the samples whose valences were -2, -1, and two were better than that of others. In contrast, the classification results of the samples whose valences were zero and one were better than that of others in the external validation classification. This may be owing to the use of SMOTE, which increases the minority class samples through oversampling, increases the number of samples with similar information to the original samples, and finally, reduces the model’s ability to classify new minority class samples. In binary and the ternary classifications, the impact of SMOTE was limited owing to the large sample size. Therefore, external validation was a further step toward verifying the model’s actual performance. Although SMOTE is suitable for large sample sizes, as the number of classes increases, the sample size of each class decreases, and its effect becomes very limited.

## Limitations

In this study, an affective quality evaluation model for multi-type urban public spaces was built. However, the proposed model had limitations in the following three aspects. First, binary and ternary classification models can be used to evaluate multiple types of public spaces. However, the results of the quinary classification were poor, and its performance could only be improved by increasing the number of samples and samples of different categories. Second, the data of emotional quality assessment could not reflect the comprehensive features of the public space because it was based on personal experience. Therefore, commercial and spatial behavior data must be added to the evaluation model to obtain detailed information about the public space. Third, human emotions include short-term and long-term effects. Users who enter a public space for the first time rely primarily on their physical senses to perceive it. After long-term use, factors such as space function, public social interaction, and place attachment become the main factors affecting evaluation. Thus, it is necessary to further analyze the long-term emotions evoked by a space to obtain a more comprehensive evaluation of its affective quality.

## Conclusions and future research

Despite the above limitations, we can confidently report that the binary and ternary affective evaluation of multiple types of spaces based on multiple physiological signals can satisfy the requirements of decision-making on urban public spaces renewal.

Whether through expert or user evaluation, the evaluation of public spaces in different regions, styles, and functions has always been a controversial problem in urban science. Our focus was on enhancing the adaptability and classification capabilities of the proposed model. To obtain a model with better adaptability, we collected data from five types of spaces in two countries to ensure the diversity of spatial data. In addition, we improved the classification performance of the model using efficient feature reduction, SMOTE algorithm, and ensemble learning. We also compared the performances of the binary, ternary, and quinary classification models. Finally, through external validation, we observed that the binary and ternary classification models outperformed the quinary model at satisfying practical requirements.

In future research, we will attempt to study the effects of long-term emotions, spatial function, and neighborhood interaction on the evaluation of spatial affective quality. Through multimodal signal extraction and new machine learning technologies, we can continuously improve the performance of the spatial quality evaluation model and provide technical support for the construction of intelligent cities.

## Supporting information

S1 Table Valence statistics of participants in 10 public spaces. (XLSX)

S2 Table Dataset of 68 signal features extracted from EDA, EMG and ECG signals. (XLSX)

S3 Table Dataset of 50 signal features after feature reduction. (XLSX)

S4 Table Dataset from eight spaces for binary classification. (XLSX)

S5 Table Data and calculation tables output after external validation. (XLSX)

S1 Fig Example of feature extraction from EDA signal. (TIF)

S2 Fig Example of feature extraction from ECG signal. (TIF)

S3 Fig Example of feature extraction from EMG signal. (TIF)

S4 Fig Data flow for training and testing a binary classification model. (TIF)

S5 Fig Data flow for training and testing a ternary classification model. (TIF)

S6 Fig Data flow for training and testing a quintuple classification model. (TIF)

S7 Fig Data flow for external validation. (TIF)

S1 Text Output of PCA. (DOCX)
