# Supplementary figures and images for: Affective computing of multi-type urban public spaces to analyze emotional quality using ensemble learning-based classification of multi-sensor data
Source: PLoS One. 2022 Jun 3;17(6):e0269176. doi: 10.1371/journal.pone.0269176 (PMC9165821; doi:10.1371/journal.pone.0269176)

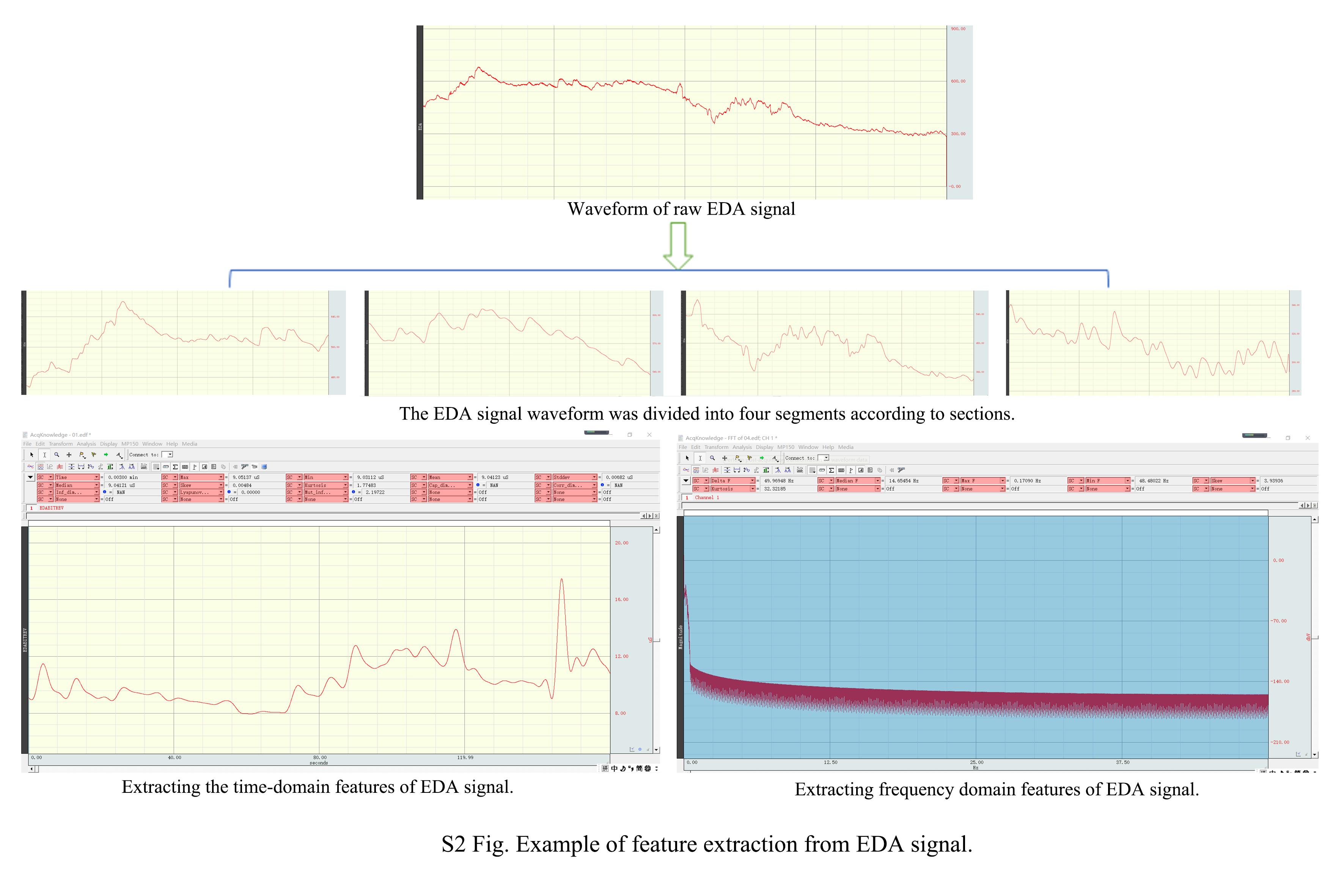

Supplement: S1 Fig — (TIF) [file pone.0269176.s006.tif]

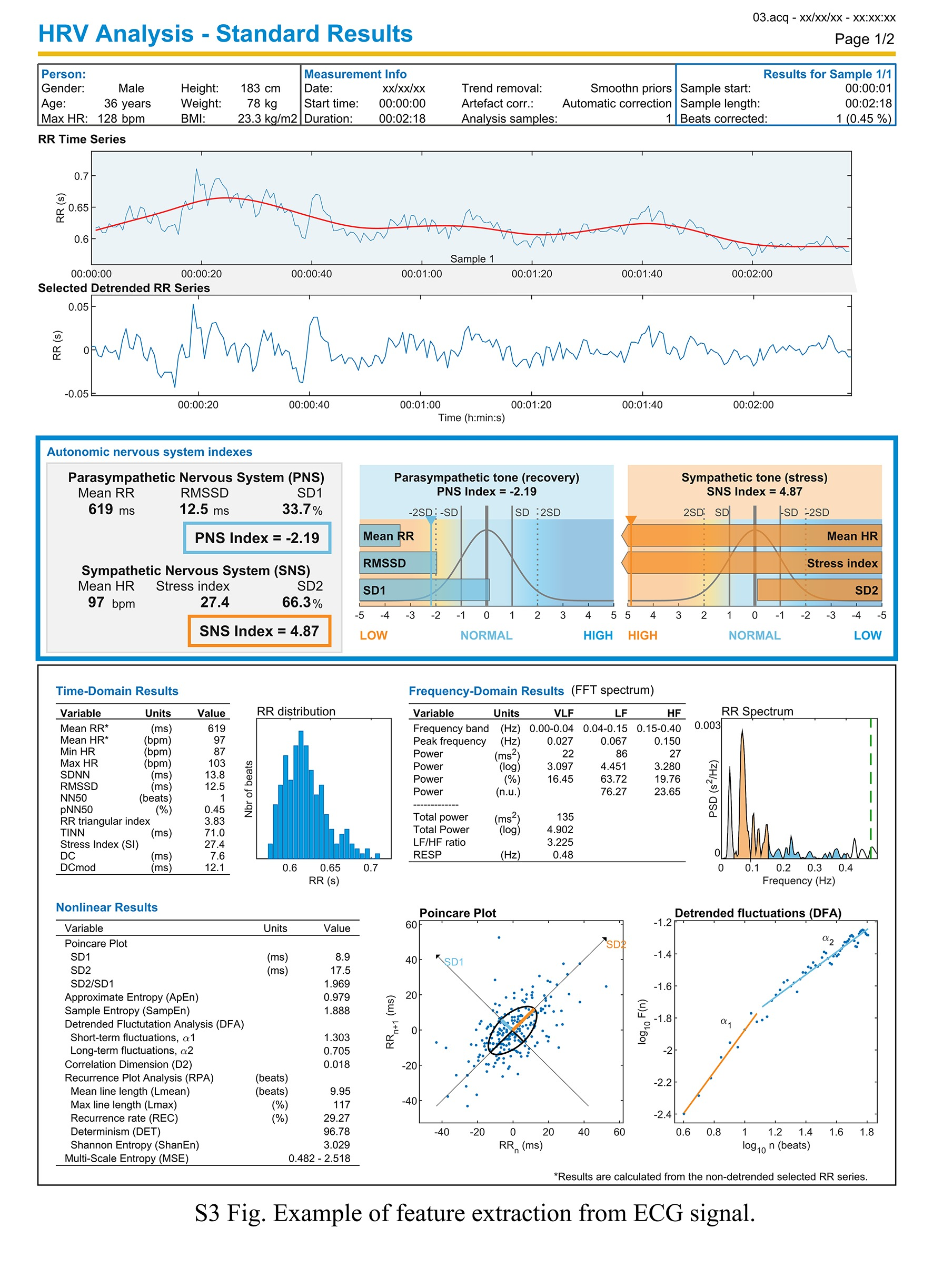

Supplement: S2 Fig — (TIF) [file pone.0269176.s007.tif]

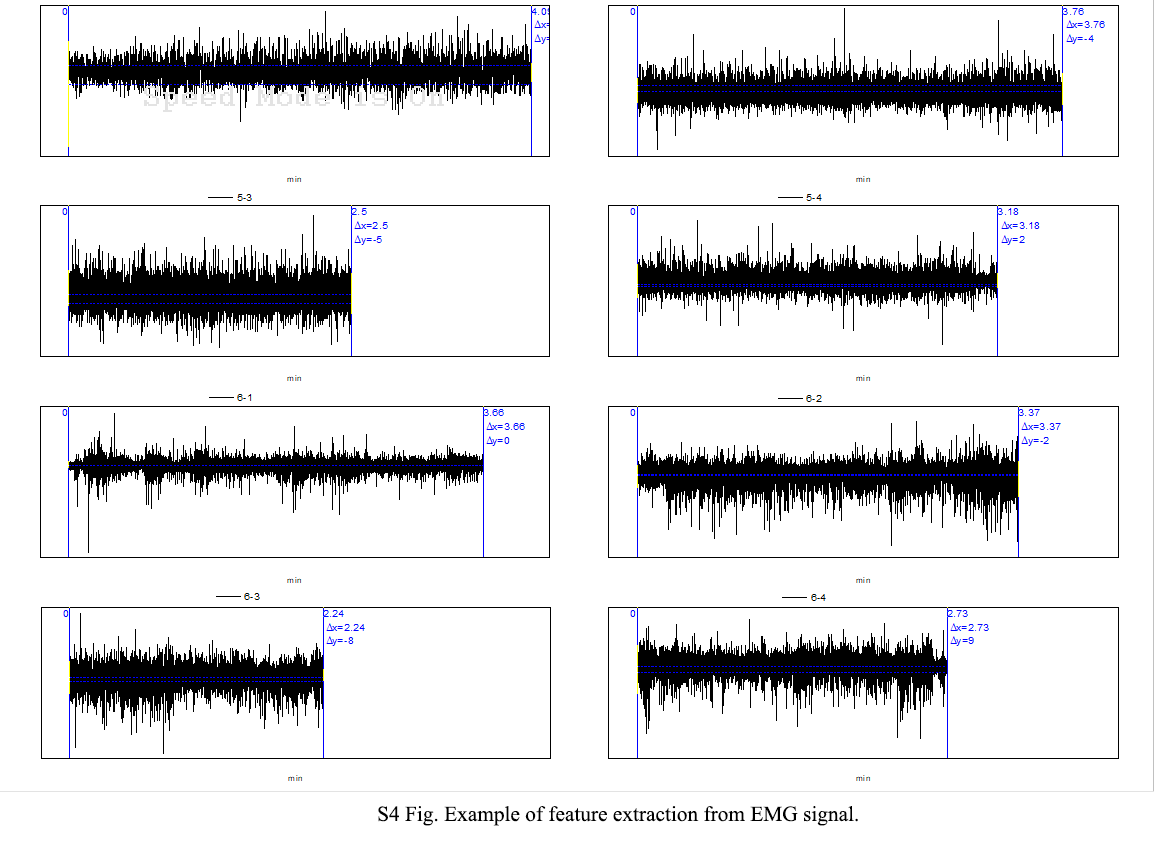

Supplement: S3 Fig — (TIF) [file pone.0269176.s008.tif]

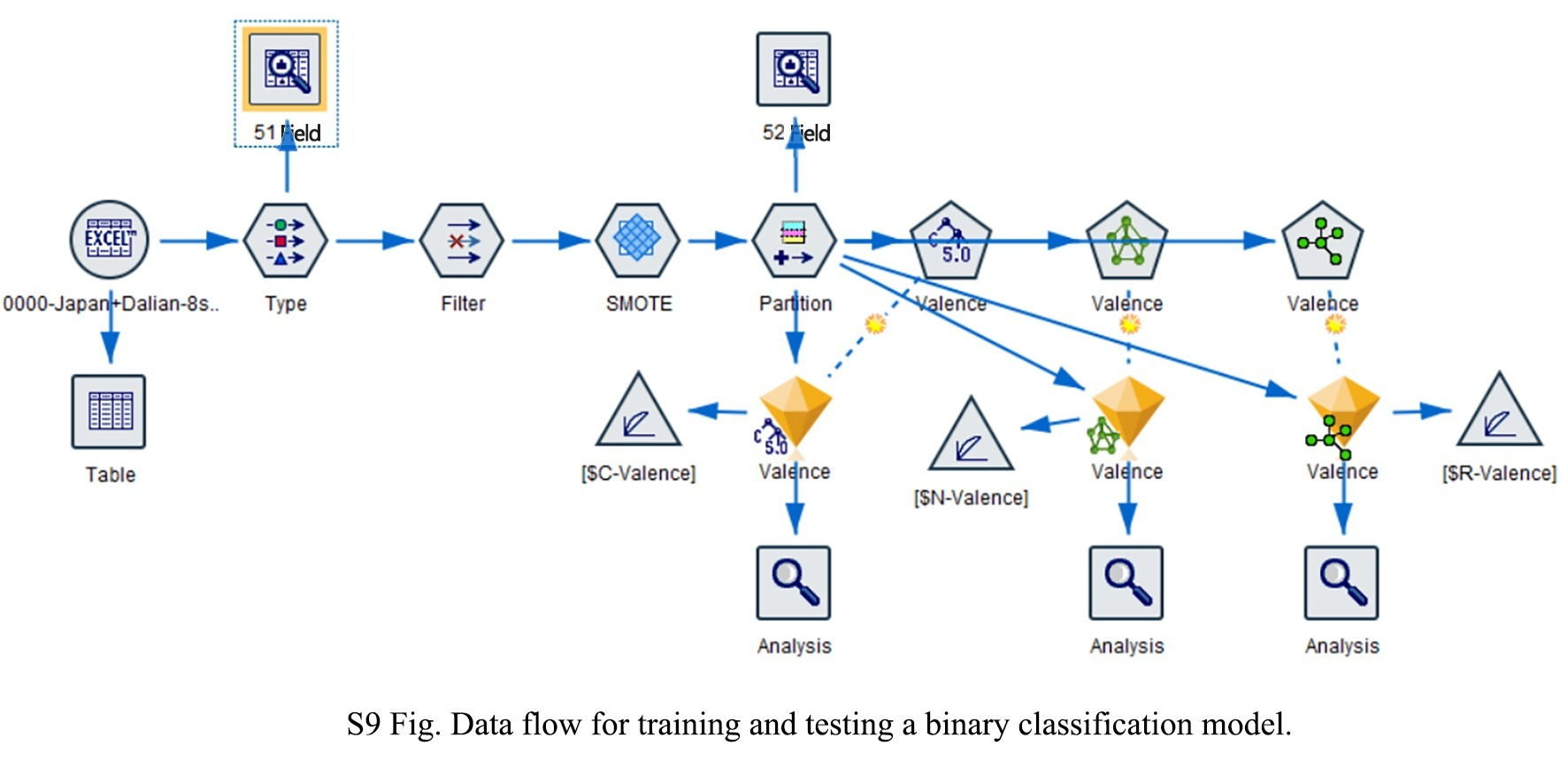

Supplement: S4 Fig — (TIF) [file pone.0269176.s009.tif]

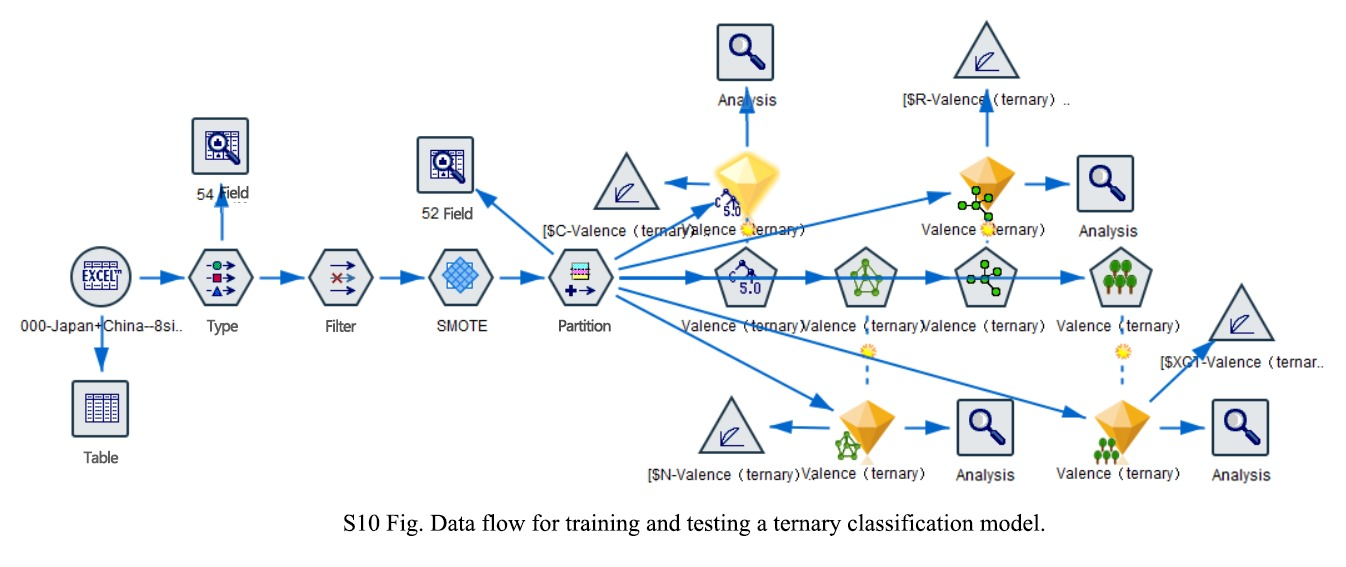

Supplement: S5 Fig — (TIF) [file pone.0269176.s010.tif]

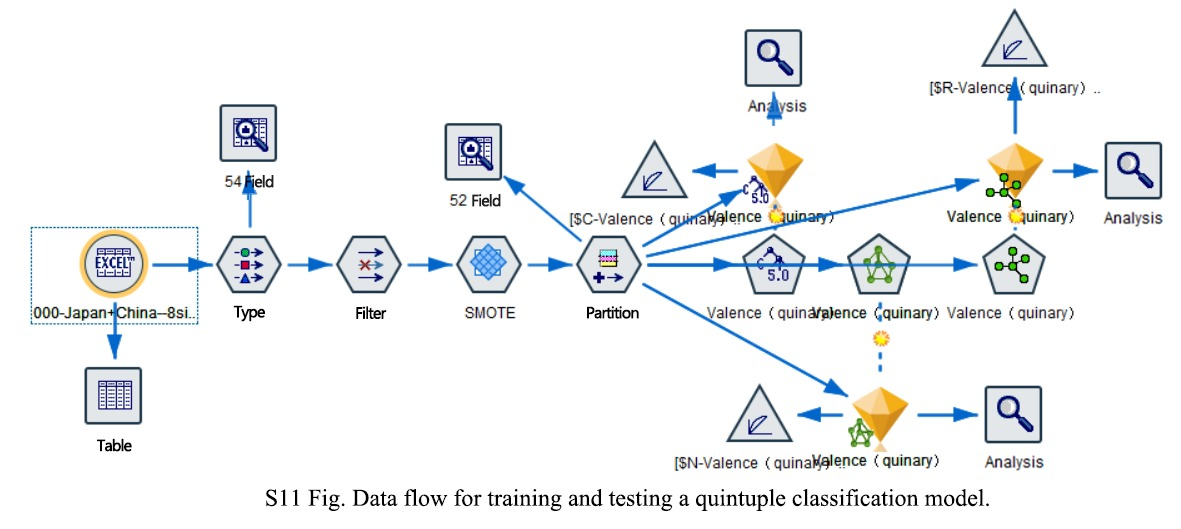

Supplement: S6 Fig — (TIF) [file pone.0269176.s011.tif]

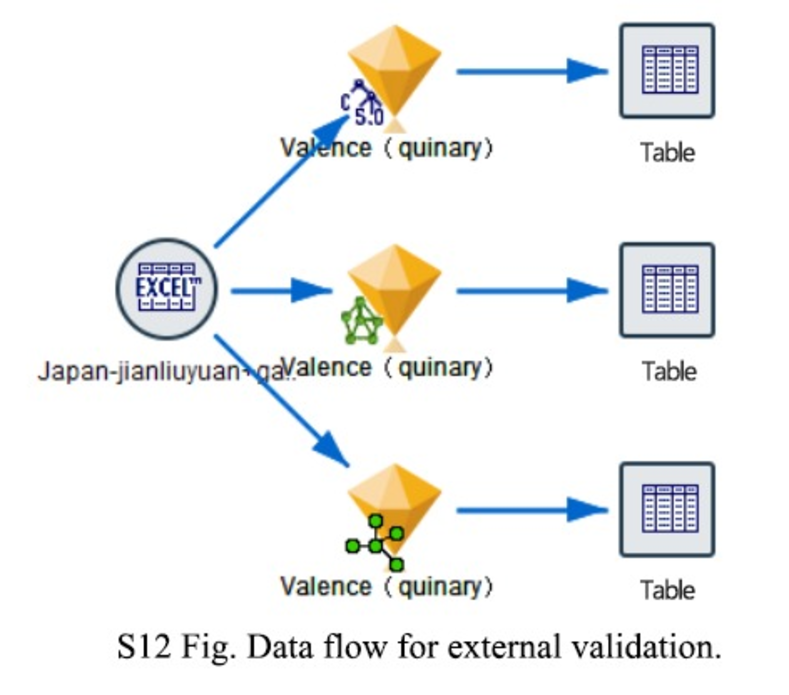

Supplement: S7 Fig — (TIF) [file pone.0269176.s012.tif]
