# Supplementary material for: Affective computing of multi-type urban public spaces to analyze emotional quality using ensemble learning-based classification of multi-sensor data
Source: PLoS One. 2022 Jun 3;17(6):e0269176. doi: 10.1371/journal.pone.0269176 (PMC9165821; doi:10.1371/journal.pone.0269176)
Supplement: S1 Text — (DOCX) [file pone.0269176.s013.docx]

The output of Principal Component Analysis (PCA)

Max（uS） Min（uS） Mean（uS） Stddev（uS） Median（uS） Skew Kurtosis Capacitydimension

Correlationdimension Informationdimension Lyapunovexponent MutualInformation MedianF（Hz） MinF（Hz）

Skew_A Kurtosis_A MeanRRms SDNNms MeanHRbeatsmin SDHRbeatsmin MinHRbeatsmin MaxHRbeatsmin RMSSDms

NNxxbeats pNNxx RRtriindex TINNms DCms DCmodms ACms ACmodms VLFHz LFHz HFHz VLFms2 LFms2 HFms2

VLFlog LFlog HFlog VLF LF HF LFn.u HFn.u Totalpowerms2 LFHFratio EDRHz SD1ms SD2ms SD2SD1ratio

ApproximateentropyApEn SampleentropySampEn alpha1 alpha2 CorrelationdimensionD2 Meanlinelengthbeats

Maxlinelengthbeats RecurrencerateREC DeterminismDET Shannonentropy Integ RMS Mean SD Max F.mean

F.med

/MISSING LISTWISE

/ANALYSIS Max（uS） Min（uS） Mean（uS） Stddev（uS） Median（uS） Skew Kurtosis Capacitydimension

Correlationdimension Informationdimension Lyapunovexponent MutualInformation MedianF（Hz） MinF（Hz）

Skew_A Kurtosis_A MeanRRms SDNNms MeanHRbeatsmin SDHRbeatsmin MinHRbeatsmin MaxHRbeatsmin RMSSDms

NNxxbeats pNNxx RRtriindex TINNms DCms DCmodms ACms ACmodms VLFHz LFHz HFHz VLFms2 LFms2 HFms2

VLFlog LFlog HFlog VLF LF HF LFn.u HFn.u Totalpowerms2 LFHFratio EDRHz SD1ms SD2ms SD2SD1ratio

ApproximateentropyApEn SampleentropySampEn alpha1 alpha2 CorrelationdimensionD2 Meanlinelengthbeats

Maxlinelengthbeats RecurrencerateREC DeterminismDET Shannonentropy Integ RMS Mean SD Max F.mean

F.med

/PRINT INITIAL CORRELATION SIG KMO EXTRACTION ROTATION FSCORE

/FORMAT SORT BLANK(0.5)

/PLOT EIGEN ROTATION

/CRITERIA MINEIGEN(1) ITERATE(25)

/EXTRACTION PC

/CRITERIA ITERATE(25)

/ROTATION VARIMAX

/SAVE REG(ALL)

/METHOD=CORRELATION.

| **KMO and Bartlett's Test** | | |
| --- | --- | --- |
| Kaiser-Meyer-Olkin Measure of Sampling Adequacy. | | .795 |
| Bartlett's Test of Sphericity | Approx. Chi-Square | 28505.035 |
|  | df | 2278 |
|  | Sig. | .000 |

| **Total Variance Explained** | | | | | | | | | |
| --- | --- | --- | --- | --- | --- | --- | --- | --- | --- |
| Component | Initial Eigenvalues | | | Extraction Sums of Squared Loadings | | | Rotation Sums of Squared Loadings | | |
|  | Total | % of Variance | Cumulative % | Total | % of Variance | Cumulative % | Total | % of Variance | Cumulative % |
| 1 | 21.253 | 31.254 | 31.254 | 21.253 | 31.254 | 31.254 | 17.620 | 25.912 | 25.912 |
| 2 | 6.655 | 9.787 | 41.041 | 6.655 | 9.787 | 41.041 | 5.494 | 8.080 | 33.992 |
| 3 | 4.839 | 7.117 | 48.157 | 4.839 | 7.117 | 48.157 | 5.394 | 7.932 | 41.924 |
| 4 | 4.575 | 6.729 | 54.886 | 4.575 | 6.729 | 54.886 | 4.694 | 6.904 | 48.827 |
| 5 | 3.642 | 5.356 | 60.242 | 3.642 | 5.356 | 60.242 | 4.447 | 6.540 | 55.367 |
| 6 | 2.960 | 4.353 | 64.595 | 2.960 | 4.353 | 64.595 | 3.537 | 5.201 | 60.568 |
| 7 | 2.684 | 3.947 | 68.542 | 2.684 | 3.947 | 68.542 | 3.129 | 4.601 | 65.170 |
| 8 | 2.547 | 3.745 | 72.287 | 2.547 | 3.745 | 72.287 | 2.834 | 4.168 | 69.337 |
| 9 | 1.673 | 2.460 | 74.747 | 1.673 | 2.460 | 74.747 | 2.176 | 3.200 | 72.538 |
| 10 | 1.549 | 2.278 | 77.025 | 1.549 | 2.278 | 77.025 | 1.825 | 2.683 | 75.221 |
| 11 | 1.368 | 2.012 | 79.037 | 1.368 | 2.012 | 79.037 | 1.602 | 2.356 | 77.577 |
| 12 | 1.222 | 1.797 | 80.834 | 1.222 | 1.797 | 80.834 | 1.492 | 2.194 | 79.771 |
| 13 | 1.173 | 1.724 | 82.558 | 1.173 | 1.724 | 82.558 | 1.386 | 2.038 | 81.810 |
| 14 | 1.127 | 1.657 | 84.215 | 1.127 | 1.657 | 84.215 | 1.356 | 1.994 | 83.804 |
| 15 | 1.065 | 1.566 | 85.781 | 1.065 | 1.566 | 85.781 | 1.344 | 1.977 | 85.781 |
| 16 | .857 | 1.260 | 87.041 |  |  |  |  |  |  |
| 17 | .832 | 1.223 | 88.264 |  |  |  |  |  |  |
| 18 | .771 | 1.134 | 89.398 |  |  |  |  |  |  |
| 19 | .761 | 1.118 | 90.516 |  |  |  |  |  |  |
| 20 | .670 | .986 | 91.502 |  |  |  |  |  |  |
| 21 | .542 | .797 | 92.299 |  |  |  |  |  |  |
| 22 | .524 | .771 | 93.069 |  |  |  |  |  |  |
| 23 | .461 | .678 | 93.747 |  |  |  |  |  |  |
| 24 | .449 | .661 | 94.408 |  |  |  |  |  |  |
| 25 | .394 | .579 | 94.987 |  |  |  |  |  |  |
| 26 | .357 | .524 | 95.511 |  |  |  |  |  |  |
| 27 | .327 | .481 | 95.993 |  |  |  |  |  |  |
| 28 | .281 | .413 | 96.405 |  |  |  |  |  |  |
| 29 | .258 | .379 | 96.785 |  |  |  |  |  |  |
| 30 | .250 | .367 | 97.152 |  |  |  |  |  |  |
| 31 | .238 | .351 | 97.502 |  |  |  |  |  |  |
| 32 | .219 | .322 | 97.824 |  |  |  |  |  |  |
| 33 | .171 | .252 | 98.076 |  |  |  |  |  |  |
| 34 | .155 | .227 | 98.304 |  |  |  |  |  |  |
| 35 | .137 | .202 | 98.505 |  |  |  |  |  |  |
| 36 | .123 | .181 | 98.686 |  |  |  |  |  |  |
| 37 | .109 | .160 | 98.846 |  |  |  |  |  |  |
| 38 | .098 | .144 | 98.990 |  |  |  |  |  |  |
| 39 | .081 | .119 | 99.109 |  |  |  |  |  |  |
| 40 | .078 | .115 | 99.224 |  |  |  |  |  |  |
| 41 | .074 | .109 | 99.333 |  |  |  |  |  |  |
| 42 | .068 | .100 | 99.432 |  |  |  |  |  |  |
| 43 | .062 | .091 | 99.524 |  |  |  |  |  |  |
| 44 | .049 | .073 | 99.597 |  |  |  |  |  |  |
| 45 | .045 | .066 | 99.663 |  |  |  |  |  |  |
| 46 | .041 | .060 | 99.723 |  |  |  |  |  |  |
| 47 | .034 | .050 | 99.773 |  |  |  |  |  |  |
| 48 | .032 | .047 | 99.820 |  |  |  |  |  |  |
| 49 | .024 | .035 | 99.856 |  |  |  |  |  |  |
| 50 | .020 | .029 | 99.884 |  |  |  |  |  |  |
| 51 | .017 | .025 | 99.909 |  |  |  |  |  |  |
| 52 | .014 | .021 | 99.930 |  |  |  |  |  |  |
| 53 | .011 | .017 | 99.947 |  |  |  |  |  |  |
| 54 | .011 | .016 | 99.963 |  |  |  |  |  |  |
| 55 | .009 | .013 | 99.976 |  |  |  |  |  |  |
| 56 | .008 | .011 | 99.987 |  |  |  |  |  |  |
| 57 | .004 | .006 | 99.993 |  |  |  |  |  |  |
| 58 | .002 | .003 | 99.996 |  |  |  |  |  |  |
| 59 | .001 | .002 | 99.998 |  |  |  |  |  |  |
| 60 | .001 | .001 | 99.999 |  |  |  |  |  |  |
| 61 | .000 | .001 | 100.000 |  |  |  |  |  |  |
| 62 | .000 | .000 | 100.000 |  |  |  |  |  |  |
| 63 | 7.974E-5 | .000 | 100.000 |  |  |  |  |  |  |
| 64 | 6.455E-5 | 9.492E-5 | 100.000 |  |  |  |  |  |  |
| 65 | 2.866E-5 | 4.215E-5 | 100.000 |  |  |  |  |  |  |
| 66 | 8.779E-7 | 1.291E-6 | 100.000 |  |  |  |  |  |  |
| 67 | 2.666E-7 | 3.921E-7 | 100.000 |  |  |  |  |  |  |

| **Component Matrix^a^** | | | | | | | | | | | | | | | |
| --- | --- | --- | --- | --- | --- | --- | --- | --- | --- | --- | --- | --- | --- | --- | --- |
|  | Component | | | | | | | | | | | | | | |
|  | 1 | 2 | 3 | 4 | 5 | 6 | 7 | 8 | 9 | 10 | 11 | 12 | 13 | 14 | 15 |
| pNNxx (%): | .964 |  |  |  |  |  |  |  |  |  |  |  |  |  |  |
| RMSSD (ms): | .964 |  |  |  |  |  |  |  |  |  |  |  |  |  |  |
| SD1 (ms): | .964 |  |  |  |  |  |  |  |  |  |  |  |  |  |  |
| DCmod (ms): | .958 |  |  |  |  |  |  |  |  |  |  |  |  |  |  |
| ACmod (ms): | -.955 |  |  |  |  |  |  |  |  |  |  |  |  |  |  |
| DC (ms): | .954 |  |  |  |  |  |  |  |  |  |  |  |  |  |  |
| HF (log): | .943 |  |  |  |  |  |  |  |  |  |  |  |  |  |  |
| AC (ms): | -.943 |  |  |  |  |  |  |  |  |  |  |  |  |  |  |
| SDNN (ms): | .942 |  |  |  |  |  |  |  |  |  |  |  |  |  |  |
| SD2 (ms): | .906 |  |  |  |  |  |  |  |  |  |  |  |  |  |  |
| TINN (ms): | .889 |  |  |  |  |  |  |  |  |  |  |  |  |  |  |
| RR tri index: | .887 |  |  |  |  |  |  |  |  |  |  |  |  |  |  |
| LF (log): | .850 |  |  |  |  |  |  |  |  |  |  |  |  |  |  |
| alpha 1: | -.793 |  |  |  |  |  |  |  |  |  |  |  |  |  |  |
| HF (%): | .787 |  |  |  |  |  |  |  |  |  |  |  |  |  |  |
| LF (n.u.): | -.781 |  |  |  |  |  |  |  |  |  |  |  |  |  |  |
| HF (n.u.): | .781 |  |  |  |  |  |  |  |  |  |  |  |  |  |  |
| NNxx (beats): | .763 |  |  |  |  |  |  |  |  |  |  |  |  |  |  |
| VLF (log): | .749 |  |  |  |  |  |  |  |  |  |  |  |  |  |  |
| SD2/SD1 ratio: | -.744 |  |  |  |  |  |  |  |  |  |  |  |  |  |  |
| HF (ms^2): | .735 |  |  |  |  |  |  |  |  |  |  |  |  |  |  |
| Total power (ms^2): | .707 |  |  |  |  |  |  |  |  |  |  |  |  |  |  |
| LF (%): | -.700 |  |  |  |  |  |  |  |  |  |  |  |  |  |  |
| Max HR (beats/min): | .661 |  |  |  |  |  |  |  |  |  |  |  |  |  |  |
| LF/HF ratio: | -.636 |  |  |  |  |  |  |  |  |  |  |  |  |  |  |
| LF (ms^2): | .580 |  |  |  |  |  |  |  |  |  |  |  |  |  |  |
| Determinism (DET) (%): | -.571 |  |  |  |  |  |  |  |  |  |  |  |  |  |  |
| Max line length (beats): |  |  |  |  |  |  |  |  |  |  |  |  |  |  |  |
| VLF (ms^2): |  |  |  |  |  |  |  |  |  |  |  |  |  |  |  |
| VLF (%): |  |  |  |  |  |  |  |  |  |  |  |  |  |  |  |
| Max |  |  |  |  |  |  |  |  |  |  |  |  |  |  |  |
| Mean HR (beats/min): |  | .625 |  |  |  |  |  |  |  |  |  |  |  |  |  |
| Shannon entropy: |  | .618 |  |  |  |  |  |  |  |  |  |  |  |  |  |
| Sample entropy (SampEn): |  | -.559 |  |  |  |  |  |  |  |  |  |  |  |  |  |
| SD |  | .559 |  | .519 |  |  |  |  |  |  |  |  |  |  |  |
| Recurrence rate (REC) (%): |  | .553 |  |  |  |  |  |  |  |  |  |  |  |  |  |
| RMS |  | .546 |  | .537 |  |  |  |  |  |  |  |  |  |  |  |
| Mean line length (beats): |  | .545 |  |  |  |  |  |  |  |  |  |  |  |  |  |
| Mean RR (ms): |  | -.535 |  |  |  |  |  |  |  |  |  |  |  |  |  |
| Information dimension |  |  |  |  |  |  |  |  |  |  |  |  |  |  |  |
| Correlation dimension |  |  |  |  |  |  |  |  |  |  |  |  |  |  |  |
| Min HR (beats/min): |  |  | -.542 |  |  |  |  |  |  |  |  |  |  |  |  |
| SD HR (beats/min): |  |  | .502 |  |  |  |  |  |  |  |  |  |  |  |  |
| Min（uS） |  |  |  | .641 |  |  |  |  |  |  |  |  |  |  |  |
| Mean（uS） |  |  |  | .627 |  |  |  |  |  |  |  |  |  |  |  |
| Median（uS） |  |  |  | .622 |  |  |  |  |  |  |  |  |  |  |  |
| Max（uS） |  |  |  | .568 |  |  |  |  |  |  |  |  |  |  |  |
| Mean |  |  |  | .542 |  |  |  |  |  |  |  |  |  |  |  |
| Integ. |  | .504 |  | .531 |  |  |  |  |  |  |  |  |  |  |  |
| Skew |  |  |  |  |  |  |  |  |  |  |  |  |  |  |  |
| Kurtosis |  |  |  |  |  |  |  |  |  |  |  |  |  |  |  |
| Capacity dimension |  |  |  |  |  |  |  |  |  |  |  |  |  |  |  |
| Kurtosis |  |  |  |  |  | -.542 |  |  |  |  |  |  |  |  |  |
| Stddev（uS） |  |  |  |  |  | .534 | -.515 |  |  |  |  |  |  |  |  |
| Mutual Information |  |  |  |  |  | .505 |  |  |  |  |  |  |  |  |  |
| Min F（Hz） |  |  |  |  |  |  |  |  |  |  |  |  |  |  |  |
| Approximate entropy (ApEn): |  |  |  |  |  |  |  | .580 |  |  |  |  |  |  |  |
| alpha 2: |  |  |  |  |  |  |  |  |  |  |  |  |  |  |  |
| LF (Hz): |  |  |  |  |  |  |  |  |  |  |  |  |  |  |  |
| F. med. |  |  |  |  |  |  |  |  |  | .648 |  |  |  |  |  |
| F. mean |  |  |  |  |  |  |  |  |  | .502 |  |  |  |  |  |
| VLF (Hz): |  |  |  |  |  |  |  |  |  |  |  |  |  |  |  |
| Correlation dimension (D2): |  |  |  |  |  |  |  |  |  |  |  |  |  |  |  |
| Median F（Hz） |  |  |  |  |  |  |  |  |  |  |  |  | -.523 |  |  |
| Skew |  |  |  |  |  |  |  |  |  |  |  |  |  |  |  |
| Lyapunovexponent |  |  |  |  |  |  |  |  |  |  |  |  |  |  |  |
| HF (Hz): |  |  |  |  |  |  |  |  |  |  |  |  |  |  |  |
| EDR (Hz): |  |  |  |  |  |  |  |  |  |  |  |  |  |  |  |

| Extraction Method: Principal Component Analysis.^a^ |
| --- |
| a. 15 components extracted. |
